# Human Keratinocytes and Fibroblasts Co-Cultured on Silk Fibroin Scaffolds Exosomally Overrelease Angiogenic and Growth Factors

**DOI:** 10.3390/cells12141827

**Published:** 2023-07-11

**Authors:** Peng Hu, Ubaldo Armato, Giuliano Freddi, Anna Chiarini, Ilaria Dal Prà

**Affiliations:** 1 Department of Surgery, Dentistry, Pediatrics & Gynecology, University of Verona Medical School, 37134 Verona, Italy; peng.hu@univr.it (P.H.); uarmato@gmail.com (U.A.); ilaria.dalpra@univr.it (I.D.P.); 2 Silk Biomaterials S.r.l., 22074 Lomazzo, Italy; giuliano@silkbiomaterials.com

**Keywords:** silk fibroin, nonwoven, scaffold design, tissue engineering, fibroblasts, HaCaT keratinocytes, biomaterial interactions, exosome-mediated signaling, angiogenic factors

## Abstract

**Objectives:** The optimal healing of skin wounds, deep burns, and chronic ulcers is an important clinical problem. Attempts to solve it have been driving the search for skin equivalents based on synthetic or natural polymers. **Methods:** Consistent with this endeavor, we used regenerated silk fibroin (SF) from *Bombyx mori* to produce a novel compound scaffold by welding a 3D carded/hydroentangled SF-microfiber-based nonwoven layer (C/H-3D-SFnw; to support dermis engineering) to an electrospun 2D SF nanofiber layer (ESFN; a basal lamina surrogate). Next, we assessed—via scanning electron microscopy, attenuated total reflectance Fourier transform infrared spectroscopy, differential scanning calorimetry, mono- and co-cultures of HaCaT keratinocytes and adult human dermal fibroblasts (HDFs), dsDNA assays, exosome isolation, double-antibody arrays, and angiogenesis assays—whether the C/H-3D-SFnws/ESFNs would allow the reconstitution of a functional human skin analog in vitro. **Results**: Physical analyses proved that the C/H-3D-SFnws/ESFNs met the requirements for human soft-tissue-like implants. dsDNA assays revealed that co-cultures of HaCaTs (on the 2D ESFN surface) and HDFs (inside the 3D C/H-3D-SFnws) grew more intensely than did the respective monocultures. Double-antibody arrays showed that the CD9^+^/CD81^+^ exosomes isolated from the 14-day pooled growth media of HDF and/or HaCaT mono- or co-cultures conveyed 35 distinct angiogenic/growth factors (AGFs). However, versus monocultures’ exosomes, HaCaT/HDF co-cultures’ exosomes (*i*) transported larger amounts of 15 AGFs, i.e., PIGF, ANGPT-1, bFGF, Tie-2, Angiogenin, VEGF-A, VEGF-D, TIMP-1/-2, GRO-α/-β/-γ, IL-1β, IL-6, IL-8, MMP-9, and MCP-1, and *(ii*) significantly more strongly stimulated human dermal microvascular endothelial cells to migrate and assemble tubes/nodes in vitro. **Conclusions**: Our results showed that both cell–cell and cell–SF interactions boosted the exosomal release of AGFs from HaCaTs/HDFs co-cultured on C/H-3D-SFnws/ESFNs. Hence, such exosomes are an asset for prospective clinical applications as they advance cell growth and neoangiogenesis and consequently graft take and skin healing. Moreover, this new integument analog could be instrumental in preclinical and translational studies on human skin pathophysiology and regeneration.

## 1. Introduction

The skin or integument isolates the body’s internal milieu from the outside environment. Being the most conspicuous of the human body’s organs (~15% of body weight in the adult), the skin has a complex three-layered structure (epidermis, dermis, and hypodermis or subcutaneous tissue); a vascular system playing relevant roles both in physiological and pathological conditions; a crucial innervation; and various annexes (glands, hair follicles, and nails). Underneath the epidermis, the dermis is a thick layer of connective tissue rich in fibroblasts, the main resident cell type, as well as macrophages, extracellular matrix (ECM), vessels, and collagen and elastic fibers. The hypodermis is rich in collagen fibers and adipose tissue. Besides storing energy, the latter favors skin’s mobility on the muscle fascial or bone periosteal planes. In brief, the skin’s complex physiological functions include (*i*) regulated epidermal self-regeneration sustained by its own stem cells; (*ii*) the impermeabilization of the body surface due to the slowly desquamating epidermal *stratum corneum*; (*iii*) thermoregulatory activity via sweat evaporation; and (*iv*) a barrier function and local immune defense against penetrating infectious agents.

Severe skin lesions significantly impact general health, mental conditions, body image, social relations, access to jobs, and healthcare costs. The current worldwide increases in the prevalence of acute skin wounds/burns and chronic ulcers (hundreds of thousands of cases per year in the United States only) have caused alarm because of the abovementioned distressing multi-level consequences [1,2]. Wounds’ healing is a quite complex set of succeeding processes (see full details in [3]). Extended (>4 cm in size) and deep skin wounds/burns and chronic (particularly diabetic) ulcers locally entail the destruction of the dermis/hypodermis vascular system and favor microbial infections. Concurring diseases, such as the abovementioned diabetes, also alter the wound’s healing process. The risk of adverse outcomes, such as hypertrophic scars/keloids, chronic wounds/ulcers, amputation, or even death, is significant [4]. To avoid such undesirable consequences, clinicians must consider the patient’s condition and use appropriate therapeutic means to properly guide the regeneration of a vascularized dermis and hypodermis, which in turn will advance epidermal regeneration.

The conventional therapeutic approach to skin injuries involves quickly closing the wound to re-establish the barrier. This is achieved by applying grafts of homologous (from the patient’s donor site), autologous (from living donors or from cadavers), or xenobiotic skin (mostly from pigs). Autologous amniotic membranes may also be grafted [5,6]. However, the needed amounts of skin grafts or amnion often exceed their actual availability, particularly in the case of burn patients. Hence, as alternatives, cultured epidermal autografts grown from tiny skin biopsies are applied to burnt areas. The latter often give uncertain results since they are applied to a vessel-devoid bed, besides entailing technical difficulties and relevant costs [7]. Skin substitutes in the form of gas-exchanging, moisture-keeping, toxicity-devoid, and infection-preventing temporary dressings are a further alternative to accelerate wound healing and prevent or mitigate hypertrophic scarring [4,8]. Typically, skin substitutes should be cheap, nontoxic, and non-inflammagenic and have good physical and mechanical features, a lengthy shelf-life, and, when necessary, regulated degradation [9,10].

In addition, the swiftly developing science of biomaterials has allowed us to produce novel types of scaffolds aimed at advancing skin tissue engineering/regeneration [8,11]. Such biomaterial scaffolds belong to two classes, being based either on a chemically synthesized polymer or on a natural biopolymer. The synthetic polymers must be mechanically adequate for the task but degradable to allow their substitution by living tissue. The most frequently used synthetic degradable polymers are poly(α-hydroxy acids), e.g., poly(glycolic acid) (PGA), poly(lactic acid) (PLA), poly-caprolactone) (PCL), and their copolymers. Poly(ethylene glycol) (PEG), poly(ethylene oxide) (PEO), polygalactose, and polyurethane have also been used with the same aim [12]. The typical benefits of artificial-compound-based scaffolds are processing ease, cheap manufacturing, good mechanical properties, controlled biodegradation rates, and potentially wide applications. Some of the commercially available skin substitutes, such as Alloderm^®^, Integra^®^, and Biobrane^®^, are composed of a combination of synthetic and biological polymers and may carry embedded fibroblasts such as Dermagraft^®^, TransCyte^®^, and the Tegaderm–nanofiber construct (see [5] for details and references). However, chemically synthesized polymers also have limitations, such as low biocompatibility, susceptibility to infection, and the release of cytotoxic and/or proinflammatory products upon degradation, altogether translating into poor performance in clinical trials.

Natural biopolymers used to produce skin substitutes include collagen, elastin, chitosan, hyaluronic acid, and silk fibroin (SF) [5,13,14]. Among the natural biopolymers, the SF protein is particularly noteworthy. It can be purified from insects such as silkworms, wasps, and beetles or from arthropods such as mites and spiders [15]. Mulberry SF (from the domesticated *Bombyx mori* silkworm) accounts for ~70–80% of the pure silk fiber mass obtained after degumming cocoons’ fibers [16]. Structurally, *Bombyx mori* SF is composed of a hydrophobic Gly-Ala-repeat-rich heavy chain (~390 kDa) and a nonrepetitive, more hydrophilic light chain (~24 kDa), joined by a disulfide bond. SF forms a stable, anti-parallel-folded molecule that, according to the ongoing physicochemical conditions, adopts three interchangeable conformations, namely Silk I, II, and III [17]. Several of SF’s characteristics meet the requirements for any biomedical material to be suitable for biomimetic scaffolds for tissue engineering [18], i.e., SF (*i*) is cheap and easy to prepare from abundant sources; (*ii*) can be highly purified, thus avoiding infectious agents’ transmission; (*iii*) allows the addition of functionally reactive groups at the side chains of its amino acids; (*iv*) does not release toxic products upon biodegradation; (*v*) has high biocompatibility and regulatable biodegradability; (*vi*) keeps its characteristics unaltered after sterilization procedures; (*vii*) has good mechanical strength and plasticity, being molded into various types of 2D or 3D structures via different physicochemical conditions, technologies, and operative modalities [19]; (*viii*) is a rather weak immunogen as significant stretches of its amino acids do occur in at least 50 human proteins [20]; and (*ix*) as-grafted 3D nonwoven scaffolds (3D-SF-nws) can guide the engineering of abundantly vascularized reticular connective tissue in living mice [21,22,23]. Regarding this rapid vascularization in vivo, we posited that the contact between the SF microfibers and the host’s connective tissue cells might induce the latter to extracellularly release angiogenic and growth factors (AGFs). It was then necessary to test our hypothesis and establish whether default cellular secretory pathways or extracellular vesicles, e.g., exosomes (Exos), were involved in the SF-induced AGFs’ release.

Exos are membrane-bound vesicles intracellularly assembled inside multivesicular bodies (MVBs). When the mature MVBs fuse with the inner surfaces of the cells’ plasma membranes, they immediately release the Exos into the extracellular matrix (ECM) [24]. Typically, Exos’ diameters range from 30 to 100 nm, thus being larger than low-density lipoproteins (LDLs) but smaller than other types of extracellular vesicles, including apoptotic bodies and blood cells. Somatic cells of any type and age incessantly produce and release Exos into the ECM. Therefore, Exos are extant in all types of body fluids, blood included, as well as in cell culture media [25]. Exos’ cargoes comprise several species of proteins, lipids, RNAs, ribosomes, and mitochondrial DNA [26]. Thus, Exos convey and deliver chemical information to both nearby and faraway cells [27]. The Exo-delivered paracrine/endocrine factors modulate the recipient cells’ proliferation, metabolic activity, and death (e.g., via apoptosis), and they impact angiogenesis/vascularization and tumor cell metastasis [28,29,30,31,32]. Hence, Exos persistently play crucial roles in physiological processes and in human diseases. Amongst other effects, the Exos released from mesenchymal stem cells (MSCs) advanced skin wound healing, which implied their potential as drug/protein delivery carriers [26,32,33,34,35].

We preliminarily tested our abovementioned hypothesis by using a novel carded/hydroentangled (C/H)-3D-SFnws seeded with either adult human dermal fibroblasts (HDFs) or adult human coronary artery smooth muscle cells (SMCs). Our results showed that, under such conditions, both cell types released Exos conveying significantly increased amounts of multiple AGFs. The same Exos powerfully induced human dermal microvascular endothelial cells (HDMVECs) cultured in vitro on a 3D gel to grow, migrate, and quickly build up lengthy tubes interconnected through numerous contacts (or nodes) [36,37].

In an early work, we set up long-term (75–95 days) mixed (or direct) co-cultures of untransformed HDFs and human keratinocytes on formic-acid-crosslinked (FA) 3D-SFnws. Thus, we showed that both cell types actively grew, established reciprocal contacts, and produced type I collagen moieties that assembled into fibers. Conversely, they did not release urea nitrogen or proinflammatory Interleukin (IL)-1β [22]. At that time, we did not check for any AGF release.

However, an ideal scaffold for skin tissue regeneration should concurrently guide the engineering of a richly vascularized dermis and hypodermis while permitting the spatially separated migration, proliferation, and differentiation of the epidermal cells and fibroblasts, and eventually wound’s closure. The said spatial separation must allow the two cell types to reciprocally exchange growth factors and other agents according to a paracrine paradigm. Therefore, to achieve these conditions, we glued a nearly flat layer of electrospun SF nanofibers (ESFNs) to one side of our most recent model of carded/hydroentangled (C/H)-3D-SFnws, the mechanical characteristics of which are akin to those of human tissue [36]. Next, we set up short-term (15 days) indirect—i.e., separated by the ESFN layer, acting as a microporous surrogate of the basal membrane—co-cultures of HaCaT keratinocytes and HDFs on the novel C/H-3D-SFnws/ESFN scaffold. The results showed that these separate yet intercommunicating HaCaT+HDF co-cultures grew most intensely while releasing Exos enriched in AGF surpluses. These Exos can effectively assist wound/burn and chronic ulcer healing. Finally, the same skin equivalent/substitute model could be used to conduct skin-related pathophysiological, pharmacological, toxicological, and cosmetological investigations.

## 2. Materials and Methods

### 2.1. Experimental Setup

**Figure 1:**
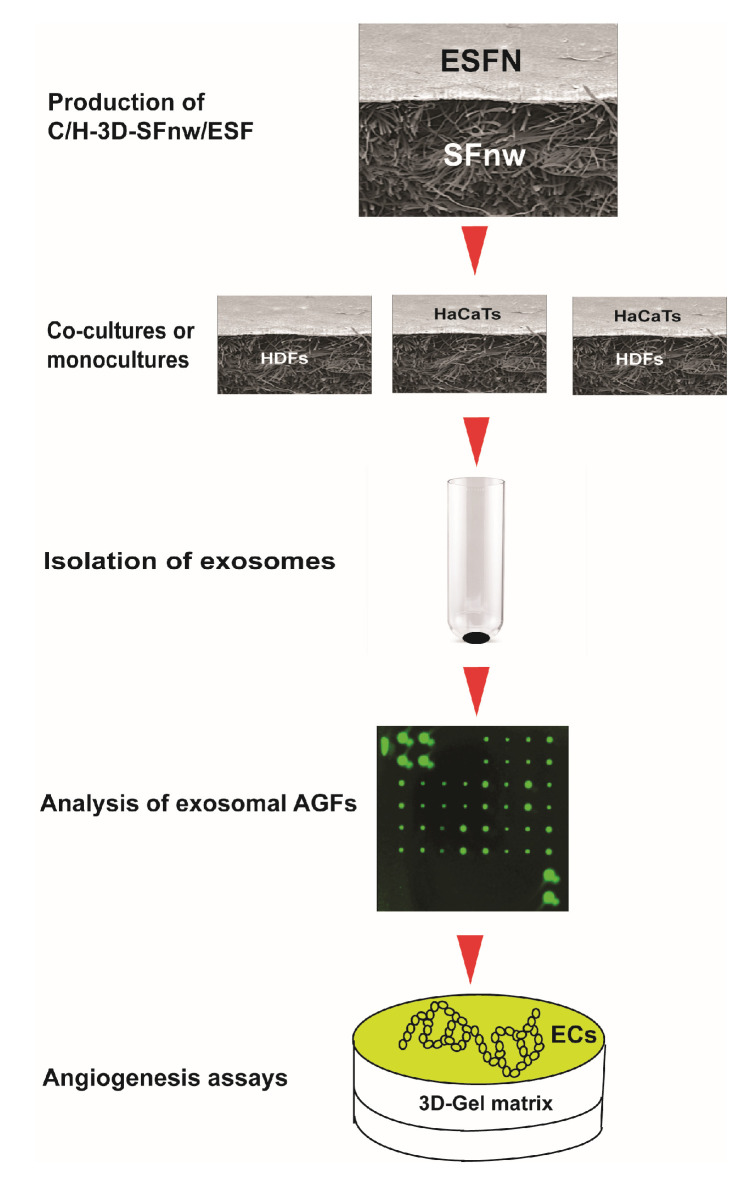
Cartoon depicting the succession of experimental procedures used in the present work from the production of the novel SF nanofiber/microfiber-based 3D nonwoven scaffolds to the angiogenesis assays of the exosomally conveyed AGFs. For details about each procedure and the respective experimental results, please consult the appropriate sections of the text.

The sequence of experimental procedures used for the present work is schematically summarized in Figure 1.

### 2.2. C/H-3D-SFnws/ESFN Composites

The C/H-3D-SFnws were produced as previously reported [36]. Briefly, sericin-deprived spun silk in staple form (average fiber length, 50 ± 7 mm) was used as a starting material. Sericin, the soluble fraction of silk protein that is suspected of being immunogenic [38], was removed by alkaline degumming, which allowed us to obtain pure SF. The staple silk underwent processing in a cotton-type flat carding machine (width, 100 cm) to obtain a carded web stabilized by mechanical hydroentanglement on both its surfaces. The novel C/H-3D-SFnws/ESFN composites were produced using electrospinning technology. A layer of electrospun SF nanofibers was deposited onto one side of the C/H-3D-SFnw, mounted on a rotating steel cylinder of 2.5 cm diameter and 25 cm length (the collector). To prepare the electrospinning dope, degummed SF fibers were dissolved for 3 h in an aqueous solution of 9.3 M lithium bromide at 60 °C, followed by dialysis against distilled water to remove salts. The use of chaotropic agents as hydrogen bond breakers is the safest method to achieve SF solubilization for medical end-uses [39]. The SF solution was cast in Petri plates and baked in a vented oven at 35 °C until complete water evaporation. The thus-obtained SF films were dissolved in FA at 8% *w*/*v* to obtain the dope. Electrospinning was performed as previously reported [40], using spinning parameters optimized for the production SF fibers with a regular sub-micrometer size: potential difference = 25 kV; flow rate = 0.8 mL h^−1^; and spinneret–collector distance = 14 cm. Coupling of the ESFN layer with the 3D-SFnw was achieved during electrospinning, according to a patented process [41]. The welding medium was a solution of the ionic liquid (1-ethyl-3-methylimidazolium acetate; EMIMAc) in water (EMIMAc/water 80/20% *v*/*v*). Thereafter, the C/H-3D-SFnws/ESFN composites were removed from the collector, flattened, and stabilized by dipping into aqueous ethanol (80% *v*/*v*), followed by overnight washing in distilled water and finally drying. The obtained composite scaffolds were further purified by microwave-assisted extraction with ethanol to remove processing aids, and then dipped into distilled water overnight and dried at room temperature.

### 2.3. Scanning Electron Microscopy (SEM)

For surface morphology analysis, C/H-3D-SFnws/ESFNs were sputter-coated with Au/Pd under a reduced-argon atmosphere in a Desk V Coating System (Denton Vacuum, Moorestown, NJ, USA) and observed under the following conditions: 10 kV acceleration voltage; 100 Å beam current; and 15 mm working distance, in a Zeiss EVO MA10 scanning electron microscope.

### 2.4. Attenuated Total Reflectance Fourier Transform Infrared Spectroscopy (ATR-FTIR)

ATR-FTIR was used to investigate the structural integrity of the C/H-3D-SFnw and ESFN components of the scaffold by evaluating the shape, position, and intensity of the prominent, conformationally sensitive amide bands of SF. An ALPHA Fourier transform infrared spectrometer (Bruker Italia Srl, Milano, Italy) equipped with an attenuated total reflectance accessory served to analyze the samples by collecting 65 scans at a 4 cm^−1^ resolution in the infrared 4000–400 cm^−1^ wavenumber range. Spectra were corrected with a linear baseline and normalized to the CH_2_ bending peak at approximately 1445 cm^−1^, a peak known to be indifferent to SF’s molecular conformation.

### 2.5. Differential Scanning Calorimetry (DSC)

DSC was used to characterize the physical properties of the C/H-3D-SFnws/ESFN scaffolds by evaluating the SF thermal transitions in a wide temperature range, from room temperature up to complete material degradation. DSC thermograms were recorded with a heat-flow differential scanning calorimeter (DSC 3500 Sirius, Netzsch, Selb, Germany). Samples (3–5 mg) were sealed in aluminum pans and subjected to a heating cycle from 50 °C to 400 °C, at a heating rate of 10 °C min^−1^, under a sweeping N2 atmosphere (flow rate of 20 mL min^−1^).

### 2.6. C/H-3D-SFnws/ESFN Samples for In Vitro Cell Cultures

After thorough washing in phosphate-buffered saline (PBS), C/H-3D-SFnws/ESFN samples were sealed in pouches under a laminar flow cabinet and finally sterilized at 55 °C for 3 h by exposing them to an ethylene oxide/CO_2_ (10/90 *v*/*v*) mixture under pressure of 42 psi (≈290 kPa) in a vacuum oven. Prior to use, the sterilized 3D-SFnws/ESFNs were routinely checked for integrity.

### 2.7. Cells

The “spontaneously immortalized aneuploid human HaCaT keratinocytes” [42] were obtained from CLS Cell Lines Service GmbH (Eppelheim, Germany) after the signing of a Material Transfer Agreement between the German Cancer Research Center (or DKFZ; Heidelberg, Germany) and the University of Verona (Verona, Italy), the Recipient Scientist being Dr. Anna Chiarini. HaCaT cells were isolated from histologically normal adult human skin samples cultured in vitro [42]. Reportedly, HaCaTs are capable of nearly full epidermal differentiation [42,43]. Since the work of Kehe et al. [44] they have been often used to model “living skin equivalents”. The seller stated that real-time PCR tests had shown that these cells were devoid of human viruses. For expansion purposes, the HaCaT cells’ culture medium was DMEM (89% *v*/*v*; Thermo Fisher Scientific, Monza, Italy) combined with heat-inactivated FBS (10% *v*/*v*; Thermo Fisher Scientific) and penicillin–streptomycin solution (1% *v*/*v*; Thermo Fisher Scientific). For the experiments, the standard FBS was changed to an Exo-depleted FBS (10% *v/v*; see below).

Nontumorigenic adult human dermal fibroblasts (HDFs) were bought from Merck Life Science S.r.l. (Milano, Italy), a supplier company of Cell Applications (San Diego, CA, USA), which certified the expression of fibronectin and the absence of human viruses, mycoplasma, bacteria, yeasts, and fungi. For initial expansion purposes, DMEM (89% *v*/*v*; Thermo Fisher Scientific), fortified with heat-inactivated (at 56 °C for 30 min) FBS (10% *v*/*v*; Thermo Fisher Scientific) and penicillin–streptomycin solution (1% *v*/*v*; Thermo Fisher Scientific), was used. For specific experiments, the standard FBS was changed to Exo-depleted FBS (10% *v*/*v*; see below).

Human dermal microvascular endothelial cells (HDMVECs) isolated from adult skin capillaries were bought from PromoCell GmbH (Heidelberg, Germany). The seller guaranteed that such cells were free from human viruses, bacteria, yeasts, fungi, and mycoplasma, and that they expressed the CD31 antigen and tested positively for Dil-acetylated low-density lipoprotein uptake. For expansion purposes, HDMVECs were grown in Endothelial Cell Growth Medium (ECGM), which included Endothelial Cell Basal Medium (ECBM; 95% *v*/*v*, No. C-22210) and a supplement mix (5% *v*/*v*, No. C-39215; all from PromoCell GmbH).

### 2.8. Exo-Depleted FBS

The experimental growth media were fortified with 10% *v*/*v* Exo-depleted FBS to ensure that the Exos under study were solely from HaCaTs and/or HDFs, i.e., with no contaminating FBS-carried Exos. To this aim, the heat-inactivated FBS was spun down twice using a centrifugal force of 100,000× *g*, each time for 120 min, in an Optima TLX ultracentrifuge (Beckman Coulter, Brea, CA, USA) equipped with a TLA 100.3 minirotor [36,45].

### 2.9. Intravital HaCaT and HDF Staining

HaCaTs and HDFs were counted using a handheld automated cell counter (Scepter^®^, Merck Life Sciences Srl, Milano, Italy), following the manufacturer’s instructions. Next, cells were suspended at a density of 1.0 × 10^6^ mL^−1^ in FBS-free medium (DMEM 99% *v*/*v*; and penicillin–streptomycin solution 1% *v/v*; Thermo Fisher Scientific) to be intravitally stained with a fluorescent lipophilic membrane dye, i.e., the red-orange fluorescent DiIC18(3) (1,1′-Dioctadecyl-3,3,3′,3′-tetramethyl-indocarbocyanine perchlorate; fluorescence excitation λ_max_ 549 nm and emission λ_max_ 565 nm; final concentration of 10 mM; Thermo Fisher Scientific), according to the seller’s instructions.

### 2.10. Mono- and Co-Cultures of HaCaTs and/or HDFs on C/H-3D-SFnws/ESFNs

For each experiment, six discs (growth area: 4.5 cm^2^) were cut from a previously sterilized C/H-3D-SFnws/ESFN. The discs were placed into as many sterile wells of a polystyrene (PS) culture plate with a lid (SPL Life Sciences, Gyeonggi-do, South Korea). Typically, when HaCaT monocultures were set up, the smooth ESFN side faced upwards, while, when HDF monocultures were set up, the C/H-3D-SFnws/ESFNs had the rough 3D-SFnw side facing upwards. Intravitally stained HaCaTs (1 × 10^5^ for each disc) were seeded and fed with 5.0 mL of culture medium (i.e., DMEM 89% *v/v*; Exo-depleted FBS 10% *v*/*v*; and 1% *v/v* penicillin–streptomycin solution; all from Thermo Fisher Scientific). The same procedure was performed for the intravitally stained HDFs. The monocultures of both types were incubated for 15 days under standard conditions (i.e., 37 °C; 95% *v*/*v* air and 5% *v*/*v* CO_2_). To set up the indirect co-cultures, 1.0 × 10^5^ intravitally pre-stained HDFs were planted onto the rough 3D-SFnw surface of each disc. Next, 5.0 mL of Exo-depleted FBS medium was added to each well. Then, cells were cultured at 37 °C in 95% *v*/*v* air, 5% *v/v* CO_2_ for 48 h. Afterwards, the C/H-3D-SFnws/ESFN discs were gently turned over, thus having their smooth ESFN sides facing upwards, and 1.0 × 10^5^ intravitally pre-stained HaCaTs were seeded onto them. Next, 5.0 mL of culture medium (DMEM 89% *v*/*v*; Exo-depleted FBS 10% *v/v*; and penicillin–streptomycin solution 1% *v*/*v*; all from Thermo Fisher Scientific) was added to each well. These co-cultures were carried out at 37 °C in a 95% *v*/*v* air, 5% *v*/*v* CO_2_ atmosphere for 15 days. In parallel, monocultures of intravitally pre-stained HaCaTs or HDFs were set up on PS culture plates for comparative purposes and cultured under the same conditions. Every 48 h, the cell-conditioned media were collected for Exos isolation and analysis.

### 2.11. Double-Strand (ds)DNA Assay

To assess the growth of monocultured or co-cultured HaCaTs and HDFs on C/H-3D-SFnws/ESFNs, their total dsDNA amounts were measured using the Pico488 (ds)DNA quantification kit (Lumiprobe GmbH, Hannover, Germany). Three specimens of each monoculture type and three of the co-culture were harvested at experimental days 3 and 15. dsDNA amounts were fluorometrically measured at an excitation λ_max_ 480 nm and an emission λ_max_ 520 nm, according to the manufacturer’s instructions and Hu et al. [37].

### 2.12. Isolation, Quantification, and Biomarkers

Each group of cell-conditioned media collected between days 3 and 15 according to Section 2.10. was thawed and pooled and its corresponding whole exosomal fraction isolated using the Total Exosome Isolation Reagent for Cell Culture Media (No. 4478359, Thermo Fisher Scientific), following the manufacturer’s protocol, as previously detailed by Hu et al. [36,37]. Total proteins of each exosomal fraction were quantified using Bradford’s protein assay dye reagent (Bio-Rad, Hercules, CA, USA). Next, the biomarker detection of the exosomal preparations was conducted using two enzyme-linked immunosorbent assay (ELISA) kits, assessing, respectively, the CD9 (ExoTEST™, HansaBio Med, Tallin, Estonia) and CD81 tetraspanins (ExoELISA-ULTRA Complete Kit, System Biosciences, LLC, Palo Alto, CA, USA). Both are typical membrane surface markers of the cell-released Exos that HaCaTs and HDFs intensely express [46,47,48].

### 2.13. Identification and Quantitation of Exo-Conveyed AGFs

The several AGFs in protein form conveyed by Exos were identified and quantified using the C-Series Human Angiogenesis Antibody Array C1000 (RayBiotech, Peachtree Corners, GA, USA), according to the manufacturer’s protocol and Hu et al. [36,37]. Briefly, equal amounts of exosomal proteins (i.e., 200 μg) from each experimental group (monocultures or co-cultures) were incubated overnight with the membrane antibody arrays, previously blocked for 1 h with Intercept^®^ PBS-blocking buffer (LI-COR Biosciences GmbH, Bad Homburg, Germany). After thorough washing cycles, the membranes were incubated for 2 h at room temperature with a mixture of array-specific biotin-conjugated primary antibodies diluted 1:250 in Intercept^®^ PBS-blocking buffer. Finally, after four washing cycles, all the membranes were incubated at room temperature for 1 h with DyLight800-conjugated streptavidin (dilution 1:7500 in Intercept^®^ PBS-blocking buffer, LGC Clinical Diagnostics’ KPL, Milford, MA, USA). The signals of the various AGFs were acquired by an Odissey^®^ scanner (LI-COR Biosciences GmbH), and their integrated intensity pixel volume (IIpv) values—expressed as the sum of the intensity values for all pixels enclosed by the feature—were quantified using the Image Studio^®^ software package (version 5.2, LI-COR Biosciences GmbH). To compare the results across multiple arrays, the IIpv values of the positive control spots from each array were used to normalize the signal responses. The results from three independent experiments were averaged and expressed as mean values ± SDs.

### 2.14. HDMVEC Migration Test

For in vitro cell migration studies, we followed the procedures of Hu et al. [36,37]. Briefly, HDMVECs were pre-labelled with fluorescent CellBrite**^®^** NIR 680 dye (1 μM; Biotium, Inc., Fremont, CA, USA) and next seeded into a silicone Culture Insert-2 Well (~10 × 10^3^ cells/well; Ibidi GmbH, Gräfelfing, Germany) kept inside a 12-well culture plate. To promote cell adhesion and the formation of a confluent monolayer, HDMVECs were cultured in ECBM fortified with 10% *v*/*v* Exo-depleted FBS for at least 24 h. Then, the Culture Insert-2 Well was removed, leaving two defined cell patches, separated by a 500-μm-wide gap. The culture medium was replaced with fresh medium (controls) or medium combined with Exos (0.5 μg mL**^−^**^1^). At 0 h, a rectangular shape (corresponding to the area of the gap) was set to define the detection zone for fluorescent signals. Next, the culture plate was incubated at 37 °C and, at different time points, the fluorescence signals due to the cells that had migrated into the gap were evaluated by means of an Odyssey**^®^** Imager and quantified using the Image Studio**^®^** software (version 5.2) (both from LI-COR Biosciences GmbH). The fluorescence intensity values detected inside the rectangular shapes were quantified in real time as the sum of the IIpv values. The HDMVECs’ migration was expressed as a percentage of the cell-covered space with respect to that of experimental 0 h. The migration assays were conducted in triplicate and the mean ± SD values were used to construct curves reflecting the time-related percentage gap reduction for each experimental group.

### 2.15. HDMVEC Tube and Node Formation Assay

The pro-angiogenic properties of the Exos released from the cells grown on C/H-3D-SFnws/ESFN scaffolds were assessed using in-vitro-cultured HDMVECs and the PromoKine Angiogenesis Assay Kit (No. PK-CA577-K905, PromoCell GmbH), according to the manufacturer’s instructions. Briefly, HDMVECs were harvested and diluted to the desired density (~1.0 × 10^5^ mL**^−^**^1^) in ECBM fortified with 2.5% *v*/*v* Exo-depleted FBS. To these cells, 0.5 µg mL**^−^**^1^ Exos released from C/HC/H-3D-SFnws/ESFN-adhering HaCaTs and/or HDFs were added. Next, 1.0 × 10^4^ HDMVECs per well were seeded onto 96-well culture plates previously coated with ECM solution (PromoCell GmbH). HDMVECs seeded on ECM gel and cultured with medium with no Exos added functioned as controls. The 96-well culture plates were incubated for 4 h at 37 °C in air with CO_2_ 5% *v*/*v*. The HDMVECs were checked every hour under an inverted microscope (IM35, Zeiss, Oberkochen, Germany). Pictures were taken with a C-P12 digital camera (OPTIKA, Ponteranica Bergamo, Italy) at 100 × magnification. The total mean tube lengths (in mm) and node numbers per microscopic field at 100 × magnification were quantified via morphometric methods [49]. The results were reported for each group as means ± SDs.

### 2.16. Statistical Analysis

Data were expressed as mean values ± SDs. Descriptive statistical analyses were conducted using the Analyse-it™ software (version 3.0) package (Analyse-it Software Ltd., Leeds, UK). The data groups’ normal distribution was checked via the Shapiro–Wilk test. Comparisons of the results from multiple experimental groups were conducted using one-way ANOVA with the post hoc Tukey’s test. A one-sided Student’s *t* test served to assess the statistical significance levels of the differences between the corresponding data from HaCaTs and HDFs co-cultured on C/H-3D-SFnws/ESFNs or monocultured on PS. Statistical significance was set at *p* < 0.05.

## 3. Results

### 3.1. C/H-3D-SFnws/ESFNs’ Morphological and Physical Characteristics

**Figure 2:**
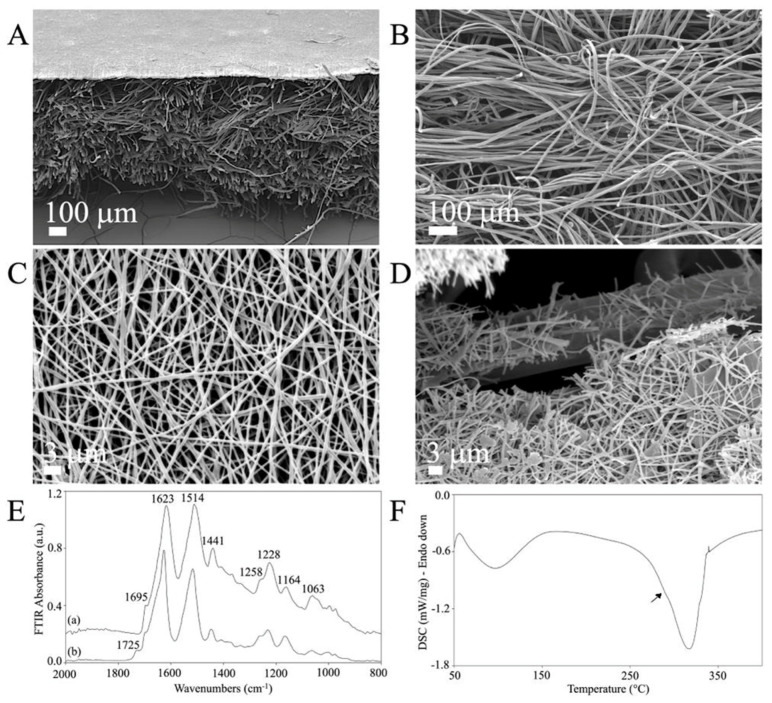
The morphology and physical characteristics of the C/H-3D-SFnws/ESFN scaffolds. (**A**–**D**): SEM images of the C/H-3D-SFnws/ESFN scaffolds. (**A**) Cross-section showing the upper thin layer of electrospun (ES) SF nanofibers welded to the surface of the microfiber-based C/H-3D-SFnw, functioning as a porous surrogate epidermis-supporting basal lamina. (**B**) The microfiber-based C/H-3D-SFnw portion of the composite, mimicking the structure of an acellular dermal matrix. (**C**) A packed network of electrospun SF nanofibers constituted the ESFN layer. (**D**) Details of a fractured area of a C/H-3D-SFnws/ESFN scaffold, revealing the interface between nanofibers and microfibers that were coupled via welding during the electrospinning procedure. (**E**) Attenuated total reflectance Fourier transform infrared spectroscopy (FTIR) spectra of the C/H-3D-SFnws/ESFN composites in the 2000 cm^−1^ to 800 cm^−1^ wavenumber range: (a) 3D-SFnw side; (b) ES side. (**F**) Differential scanning calorimetry (DSC) thermogram of the C/H-3D-SFnws/ESFN composites, with a prominent melting/degradation peak at 317 °C, corresponding to the C/H-3D-SFnw component, and a shoulder at approximately 290 °C (arrow), representing the contribution of the ESFN component.

Low-magnification SEM cross-section images revealed the double structure of the novel C/H-3D-SFnws/ESFN scaffold (Figure 2A). The original C/H-3D-SFnws were obtained by carding/hydroentangling SF fibers of 50 ± 7 mm length and 10–14 μm diameter. The nonwoven layers were 0.52 ± 0.3 mm thick (determined after UNI EN ISO 5084:1998) and weighed 58 g/m^2^ (Figure 2B). Coupling with the ESFN resulted in a composite scaffold with an overall average thickness of 0.82 ± 0.07 mm. The electrospun SF nanofibers were homogeneous in size (average diameter in the 400–600 nm range), with pores of 3 μm mean diameter (Figure 2A,C). During electrospinning, one of the ESFN surfaces was welded to the bulkier, microfiber-based, highly porous structure of the C/H-3D-SFnws. Together, the welded structures were intended to function as an acellular dermal matrix of SF microfibers coupled with a surrogate basal lamina of SF nanofibers supporting the regenerating epidermal keratinocytes (see Figure 2A and details at the fractured surface in Figure 2D).

The C/H-3D-SFnws/ESFNs’ physical properties were characterized via ATR-FTIR and DSC.

The ATR-FTIR results showed the typical attenuated total reflectance from the 2000 cm^−1^ to 800 cm^−1^ wavenumber range of the new scaffold’s samples. This spectral pattern is SF’s fingerprint, as it exhibits multiple absorption bands that are heavily influenced by its physical and chemical structure [50,51]. As mentioned above, the C/H-3D-SFnws/ESFNs had two wholly distinctive components represented by SF micro- and nanofibers, respectively. As a result, each component featured its own set of data (Figure 2E). Concerning the 3D-SFnws, the bands of Amide I at 1623 cm^−1^, with a shoulder at 1695 cm^−1^; Amide II at 1514 cm^−1^; and Amide III at 1228 cm^−1^, with a shoulder at approximately 1258 cm^−1^, were all typical of the β-sheet molecular conformation of crystalline and oriented SF microfibers. Regarding the ESFN side, the band at 1725 cm^−1^ was typical of the electrospun SF nanofibers, likely due to FA residues such as SF-bound formate [52].

On the other hand, the DSC thermogram of the C/H-3D-SFnws/ESFN composite showed a prominent melting/degradation peak at 317 °C and a shoulder at approximately 290 °C (arrow), representing the contribution of the ESFN layer to the degradation of the scaffolds, which is typical of SF fibers endowed with a high degree of molecular order and crystallinity (Figure 2F) [51].

### 3.2. Cultures of HaCaTs and/or HDFs on C/H-3D-SFnws/ESFN Scaffolds

#### 3.2.1. HDF Monocultures

**Figure 3:**
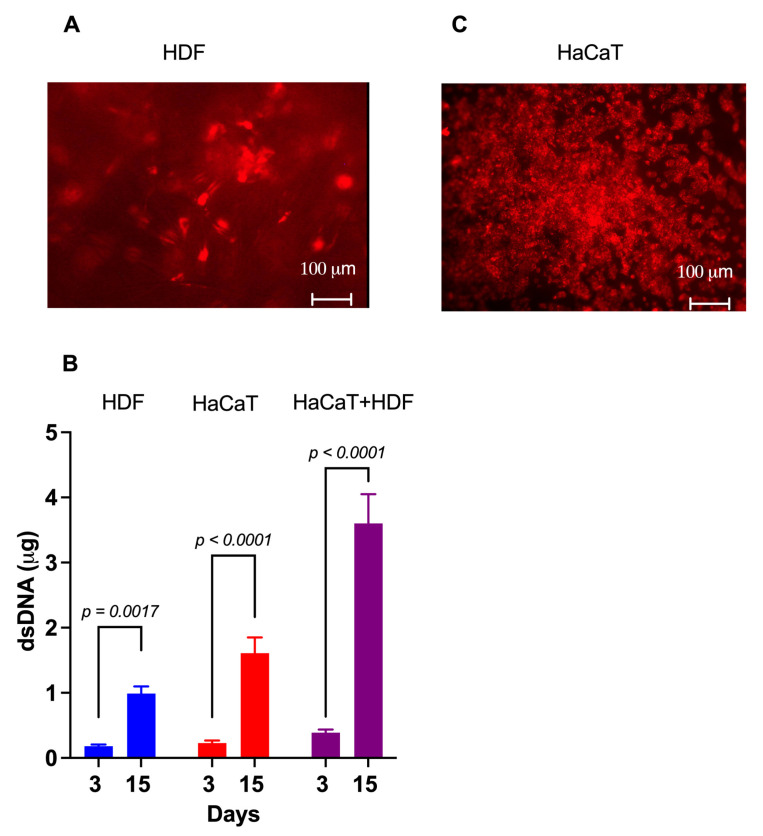
The growth of HaCaTs and HDFs on C/H-3D-SFnws/ESFN scaffolds. (**A**) When viewed under the fluorescent microscope at λ_max_ 565 nm, most of the intravitally stained HDFs that stuck to the scaffold’s 3D-SFnw side emitted a red fluorescence and appeared to be on different focal planes. Conversely, the SF microfibers were not visible as they emitted no interfering fluorescence at λ_max_ 565 nm. (**B**) The significant increases between day 3 and day 15 in vitro in the dsDNA amounts attached to the C/H-3D-SFnws/ESFN scaffolds (HaCaTs on the ESFN side; HDFs on the C/H-3D-SFnw side). These data indicate the intensity of the cells’ growth under the conditions studied. dsDNA assays were conducted as described in the Section 2. Each bar is the mean value ± SD of three different duplicate determinations for any time point. A one-sided Student’s *t* test was used to conduct the statistical analysis. (**C**) After seeding, HaCaTs attached to and grew on the ESFN side of the C/H-3D-SFnws/ESFN scaffolds. The intravitally stained HaCaTs emitted a red fluorescence at λ_max_ 565 nm. In spite of the much more intense crowding of the SF nanofibers as compared to the 3D-SFnw microfibers, the ESFN layer did not emit any interfering autofluorescence at λ_max_ 565 nm.

Within 4 h of careful planting, approximately 85% of the intravitally labelled HDFs had adhered to the rough C/H-3D-SFnw side of the scaffolds. Microscopic observations corroborated the numerical rise in the HDFs over time while they individually adhered to the SF microfibers and progressively invaded and colonized the intervening voids (Figure 3A). Between experimental days 3 and 15, the HDFs’ growth corresponded to a 4.7-fold rise (*p* = 0.0017) in the amount of (ds)DNA associated with the C/H-3D-SFnws/ESFN scaffolds (Figure 3B).

#### 3.2.2. HaCaT Monocultures

Four hours after careful seeding, 80–90% of the intravitally stained HaCaTs had adhered to the ESFN surfaces of the C/H-3D-SFnws/ESFN scaffolds. HaCaTs numerically increased over time while clustering together to form confluent islands or colonies of packed cells (Figure 3C). Between days 3 and 15 in culture, the HaCaTs’ growth corresponded to a 6.5-fold increase (*p* < 0.0001) in the dsDNA amount bound to the C/H-3D-SFnws/ESFNs (Figure 3C). At variance with HDFs, which had ample 3D spaces at their disposal and kept growing, HaCaT keratinocytes stopped increasing in number (i.e., in dsDNA amount) after reaching confluence at the 11th day in culture. This finding is in line with the results reported by Lemaître et al. [53].

#### 3.2.3. HaCaT+HDF Indirect Co-Cultures

The rather thick ESFN layer of the C/H-3D-SFnws/ESFN scaffolds did not allow us to simultaneously observe both co-cultured cell types under the inverted fluorescent microscope. However, the results of the dsDNA assays showed that between days 3 and 15, the total amount, i.e., HaCaT+HDF, of the scaffold-attached dsDNA increased 9.8-fold (*p* < 0.0001; Figure 2B). Notably, this same dsDNA amount exceeded by +37.3% (*p* < 0.05) the sum of the dsDNA amounts observed at day 15 in the respective monocultures on the same scaffolds. This finding indicated that growth-promoting interactions had occurred between the co-cultured HDFs and HaCaTs [54,55,56]. Therefore, it was worth investigating whether the content of their respectively released Exos had any angiogenic/growth stimulatory potential.

### 3.3. AGF-Loaded Exos Released from Co-Cultures and Monocultures of HaCaTs and/or HDFs on C/H-3D-SFnws/ESFNs

#### 3.3.1. AGF-Related Total IIpv Values of Double-Antibody Array Membranes

**Figure 4:**
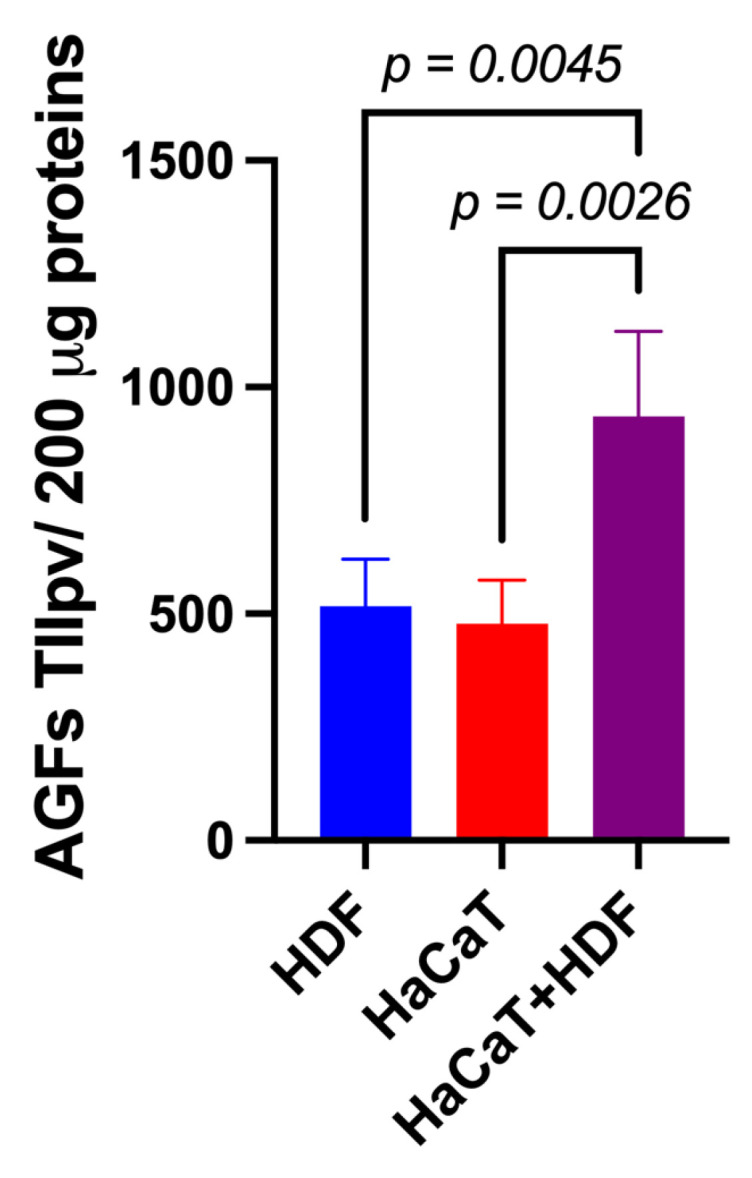
The indirect (i.e., with an interposed porous ESFN layer) HaCaT+HDF co-cultures released via Exos a significantly larger total amount of AGFs than did the corresponding monocultures on the same C/H-3D-SFnws/ESFN scaffolds. Cell cultures, 3 to 15 days after exosome isolation and pooling, double-antibody arrays challenged with equal exosomal protein amounts (200 μg) from each different experimental group, and total integrated intensity pixel volume (TIIpv) values determinations were conducted as detailed in the Section 2. Bars are TIIpv mean values ± SD from three separate experiments, each conducted in duplicate. Numbers shown are original values × 10^3^. ANOVA with post hoc Tukey’s test was used to conduct the statistical analysis.

Using specific array membranes challenged with equal amounts (i.e., 200 μg) of exosomal proteins released from each of the three experimental groups considered (i.e., HaCaT+HDF co-cultures; HaCaT monocultures; and HDF monocultures), we identified the respectively conveyed AGFs and determined the IIpv values pertaining to each AGF-specific spot. Notably, after challenging with the exosomal proteins and developing the array membranes, all the duplicate spots related to a total of 35 distinct AGFs reacted positively (Figure S1). Interestingly, under identical conditions, the total AGF-related IIpv values of the array membranes challenged with Exos released from HaCaT+HDF co-cultures were significantly higher than those of the membranes exposed to equal amounts of Exos from HaCaT or HDF monocultures (Figure 4).

These findings indicated that the interactions between HaCaTs and HDFs indirectly co-cultured on the SF-made scaffold advanced the exosomal release of total AGFs as compared to that from HDF or HaCaT monocultures on the same scaffold.

#### 3.3.2. SF-HaCaT+HDF-Exos Conveyed Greater Amounts of AGFs Than SF-HaCaT-Exos and SF-HDF-Exos

**Figure 5:**
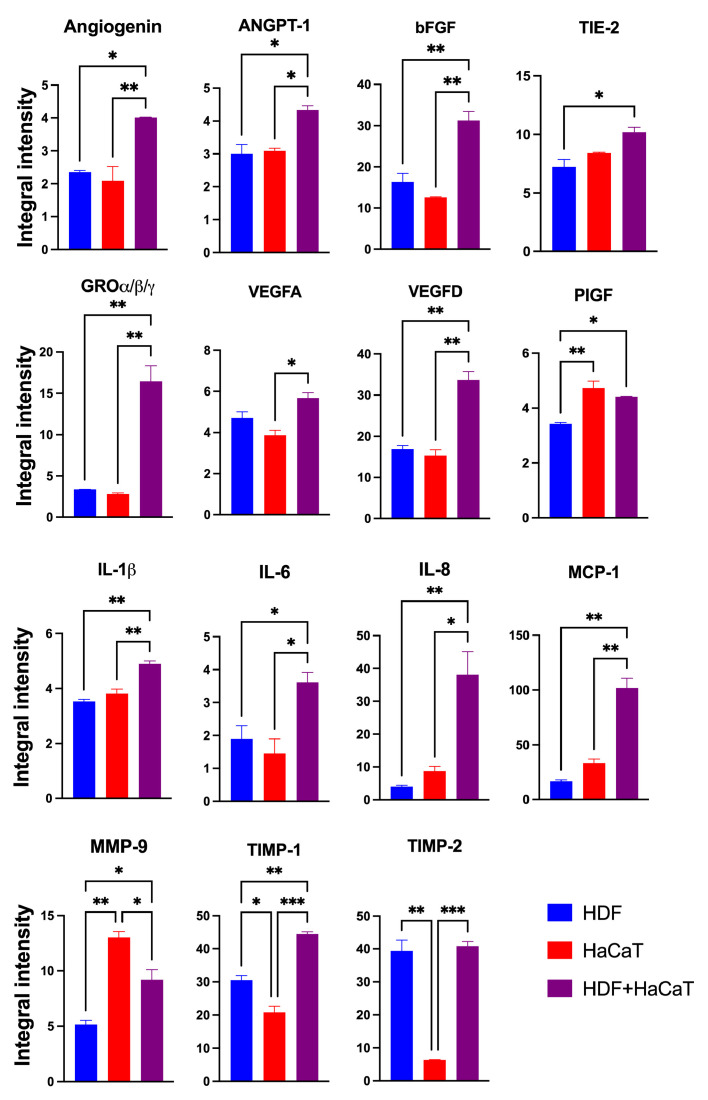
The significantly increased IIpv mean values of the 15 AGFs conveyed by the pooled (3-to-15-day) SF-HaCaT+HDF-Exos as compared to those transported by the SF-HaCaT-Exos and SF-HDF-Exos. HaCaTs and/or HDFs were co-cultured or monocultured on C/H-3D-SFnws/ESFN scaffolds, their released Exos isolated and pooled, and the AGFs present in equal amounts (200 μg) of exosomal proteins identified and quantified by means of specific double-antibody protein arrays, as detailed in the Section 2. The bars are IIpv mean values ± SD from 3 distinct experiments, each conducted in duplicate. Values shown are original numbers × 10^3^. ANOVA with post hoc Tukey’s test was used for the statistical analyses. Levels of indicated statistical differences are *, *p* < 0.05; **, *p* < 0.008; ***, *p* < 0.001.

The Exos released from the HaCaT+HDF co-cultures (henceforth named SF-HaCaT+HDF-Exos) and from each cell type’s monocultures (henceforth named SF-HDF-Exos and SF-HaCaT-Exos, respectively) on C/H-3D-SFnws/ESFNs conveyed all 35 AGFs (in protein form) that the array could identify. Most importantly, the SF-HaCaT+HDF-Exos transported a group of 15 AGFs in significantly (*p* < 0.05) larger amounts than did the SF-HDF-Exos or SF-HaCaT-Exos. Comparatively, the most remarkable AGF increases pertained to Angiogenin, bFGF, GRO-α/-β/-γ, VEGF-D, IL-6, IL-8, MCP-1, and TIMP-1. The increases in ANGPT-1, TIE-2, VEGF-A, IL-1β, and PIGF were smaller yet still significant. TIMP-2 levels were alike in SF-HDF-Exos and SF-HaCaT+HDF-Exos, but much higher than those in SF-HaCaT-Exos. Finally, the MMP-9 levels of SF-HaCaT+HDF-Exos were lower than those in SF-HaCaT-Exos but higher than those in SF-HDF-Exos (Figure 5). The remaining AGFs were conveyed in alike (*p* > 0.05) amounts by the three groups of Exos considered (Figure S1). Hence, the paracrine interactions between indirectly co-cultured HDFs and HaCaTs significantly increased the exosomal loads of 15 AGFs as compared to those of the HDF and HaCaT monocultures on the same C/H-3D-SFnws/ESFNs. Abundant evidence has proven that the above increased AGFs exert significant growth- and angiogenesis-promoting effects on skin tissue components (see, for details, Table 1) [57,58,59,60,61,62,63,64,65,66,67,68,69,70,71,72,73,74,75,76,77,78,79,80,81,82,83,84,85,86,87,88,89,90,91,92,93,94,95,96,97,98,99,100,101,102,103,104,105,106,107,108,109,110,111,112,113,114,115,116,117,118,119,120,121,122,123].

However, did the same cells’ attachment to the SF-based substrate also drive at least part of the increase in the exosomal loads of specific AGFs? To answer this question, we compared the exosomal AGF content of monocultures of HaCaTs and HDFs on two different substrates, i.e., C/H-3D-SFnws/ESFNs and polystyrene (PS).

#### 3.3.3. SF-HaCaT-Exos Conveyed Greater Amounts of AGFs Than PS-HaCaT-Exos 

**Figure 6:**
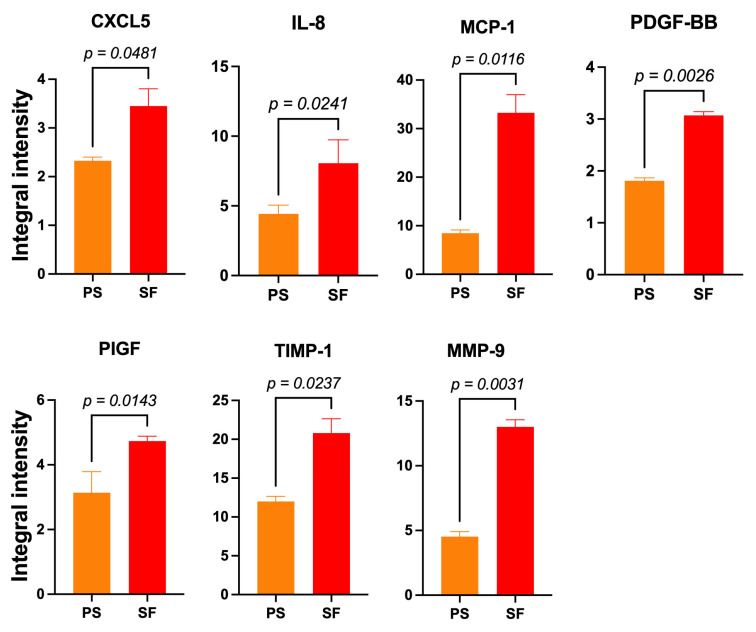
The significantly increased mean IIpv values of 7 AGFs that the pooled (3-to-15-day) SF-HaCaT-Exos conveyed as compared to those that PS-HaCaT-Exos transported. The same amounts (200 μg) of exosomal proteins from the two monoculture groups served to challenge specific membrane-based double-antibody arrays. Bars are mean IIpv values ± SD from three distinct experiments, each conducted in duplicate. Numbers shown are the original values × 10^3^. One-sided Student’s *t* test was used for statistical analyses. For more technical details, see the Section 2.

The pooled 3-to-15-day SF-HaCaT-Exos conveyed significantly (*p* < 0.05) larger amounts of seven AGFs, i.e., CXCL5 (1.48-fold), IL-8 (1.78-fold), MCP-1 (3.93-fold), PDGF-BB (1.7-fold), PIGF (1.48-fold), TIMP-1 (1.73-fold), and MMP-9 (2.87-fold) (Figure 6), than did the PS-HaCaT-Exos. Therefore, the HaCaTs’ adhesion to the ESFN substrate particularly enhanced the exosomal loading and transport of the abovementioned AGFs. Table 1 lists the main angiogenesis-/growth-promoting effects of these AGFs on skin constituents (*q.v*.). Conversely, the PS-HaCaT-Exos conveyed a significantly larger amount (1.53-fold) of the chemokine CCL1 (small inducible cytokine A1 or I-309 in humans) than the SF-HaCaT-Exos did.

#### 3.3.4. SF-HDF-Exos Conveyed Greater Amounts of AGFs Than PS HDF-Exos

Compared to previously published results (see, for details, [36]), the SF-HDF-Exos from cells adhering to the SFnw portion of the new scaffold conveyed significantly (*p* < 0.05) greater amounts of 10 AGFs—i.e., ANGPT-1 (1.5-fold), ANGPT-2 (1.5-fold), TIE-2 (1.3-fold), GRO-α/β/γ (1.6-fold), uPAR (1.5-fold), IL-1α (1.5-fold), IL-4 (1.4-fold), IL-8 (2.2-fold), MMP-1 (1.3-fold), TIMP-1 (2.9-fold).

Table 1 summarizes the relevant growth- and angiogenesis-stimulatory properties of the AGFs released from keratinocytes and/or fibroblasts on skin tissue constituents.

Altogether, these observations showed that, besides the intercellular paracrine communications occurring in the HaCaT+HDF co-cultures, the cells’ attachment to the SF substrate played, by itself, a role conducive to increases in the amounts of AGFs released via Exos from both cell types.

#### 3.3.5. SF-HaCaT+HDF-Exos, SF-HaCaT-Exos, and SF-HDF-Exos Significantly Stimulated HDMVECs’ Migration and Tube/Node Formation

Finally, we assessed whether the AGFs conveyed by the Exos released from co-cultures and monocultures of HaCaTs and HDFs on C/H-3D-SFnws/ESFNs exerted any notable proangiogenic effects on HDMVECs in vitro. As parallel controls, we used untreated (no Exos added) HDMVECs.

**Figure 7:**
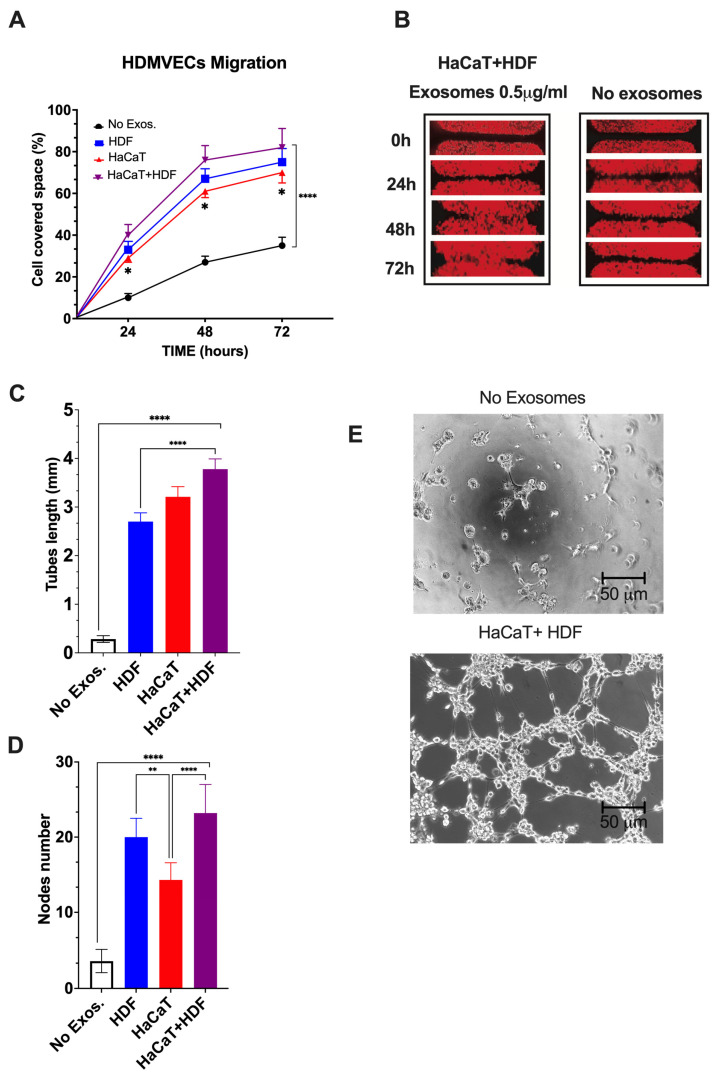
The SF-HaCaT+HDF-Exos’, SF-HaCaT-Exos’, and SF-HDF-Exos’ effects on HDVEMECs’ migratory (**A**,**B**) and proangiogenic (**C**–**E**) activity as compared to those of non-Exo-exposed (control) HDVEMECs. (**A**) Percentage gap space values covered within 72 h by migrating HDMVECs, either untreated or treated with Exos (0.5 μg mL^−1^) from the indicated sources. Triplicate results were averaged, and the dots are the mean values ± SD. ANOVA and post hoc Tukey’s test were used to conduct the statistical analysis. *, *p* < 0.02 at least between SF-HaCaT-Exos and SF-HaCaT+HDF-Exos. ****, *p* < 0.0001 between non-Exo-exposed samples (controls) and each of the three indicated treatments. (**B**) A typical example of the time-related stimulation of HDMVEC migration into the gap space by a single dose (0.5 μg mL^−1^) of SF-HaCaT+HDF-Exos vs. parallel controls (no Exos added). (**C**,**D**) The total lengths (in mm) of newly formed endothelial tubes (**C**) and the corresponding numbers of nodes (**D**) per microscopic field under the conditions of the tests, i.e., (*i*) no Exos, control HDMVECs (no Exos added) and (*ii*) HDF, HaCaT, HaCaT+HDF, HDMVECs exposed to a single dose (0.5 μg mL-1) of Exos released from each of the indicated experimental groups. Both in (*i*) and (*ii*), HDMVECs were cultured in vitro on ECM gel. The number of nodes and the total tube length (in mm) per microscopic field of 332,667 μm^2^ area were assessed via morphometric methods [49] using pictures taken at 100× magnification of 30 microscopic fields for each exosomal treatment. Triplicate results were averaged, and the bars show the mean values ± SD. ANOVA and post hoc Tukey’s test were used for statistical analysis. ****, *p* < 0.0001. **, *p* < 0.001. (**E**) Examples of phase contrast microscopic pictures showing that the untreated (no Exos added) HDMVECs formed very few endothelial tubes and nodes, while SF-HaCaT+HDF-Exo-treated (0.5 μg mL^−1^) HDMVECs produced, within 4 h, abundant tubes and nodes. In either instance, HDMVECs were cultured on ECM gel. Bars are equal to 50 μm. For further technical details, please consult the Section 2.

HDMVECs’ migration. As compared to controls, the migratory activity into the “wound area” of HDMVECs exposed to a range of doses (i.e., 0.5 to 5.0 μg mL^−1^) of SF-HaCaT+HDF-, SF-HaCaT-, or SF-HDF-Exos increased significantly (*p* < 0.0001). The intensities of the observed stimulatory effects were independent of the dose in the range tested. The elicited effects were maximal during the first 48 h, thereafter tending to plateau (Figure 7A,B).

As a typical example, a single dose (0.5 μg mL^−1^) of the SF-HaCaT+HDF-Exos induced, by 72 h, the HDMVECs to cover a portion of the gap’s area that was 3.1-fold greater (*p* < 0.0001) than that overlain by the untreated HDMVECs. On the other hand, a single dose (0.5 μg mL^−1^) of SF-HDF-Exos exerted slightly less intense promigratory effects, which statistically did not differ (*p* = 0.2218) from those brought about by SF-HaCaT+HDF-Exos. Conversely, at the same dose (0.5 μg mL^−1^), the SF-HaCaT-Exos elicited the least intense promigratory effects on HDMVECs, which significantly (*p* = 0.0101) differed from those brought about by the SF-HaCaT+HDF-Exos (Figure 7A,B). Finally, the mobilization of the Exo-treated HDMVECs in directions other than the gaps was also clearly discernible in all the groups tested.

HDMVECs’ tube and node formation. Control (no Exos added) HDMVECs plated on ECM gel in a medium with 5% *v/v* Exo-depleted FBS assembled quite a few tubes and nodes (Figure 7C,E). Conversely, within the range of doses tested (i.e., 0.5 to 5.0 μg mL^−1^ exosomal proteins), the intensities of the angiogenic effects were alike (*p* > 0.05), i.e., dose-independent for each group tested [36,37]. Thus, as an example, the treatment with a dose of 0.5 μg mL^−1^ of SF-HaCaT+HDF-Exos increased 15.1-fold the tube length and 6.8-fold the number of nodes per microscopic field vs. controls (*p* < 0.0001 in either instance) (Figure 7C–E). Moreover, the same dose (0.5 μg mL^−1^) of SF-HaCaT+HDF-Exos more intensely increased both HDMVECs’ mean tube lengths (+66.7%) and mean node numbers (+76.0%) per microscopic field than did equal amounts of the SF-HDF-Exos and the SF-HaCaT-Exos (*p* < 0.001 at least in either instance) (Figure 7C–E).

On the other hand, a single dose (0.5 μg mL^−1^) of SF-HDF-Exos elicited a 10.7-fold increase in HDMVECs’ tube lengths and a 5.9-fold rise in node number per microscopic field vs. untreated (no Exos added) cells (*p* < 0.0001 in either instance). These effects were weaker than those of the same single SF-HaCaT+HDF-Exo dose as regards the endothelial tube length (−29.0%, *p* < 0.0001), while the increases in node numbers were similar (*p* > 0.05) (Figure 7C–E). Conversely, a single dose (0.5 μg mL^−1^) of SF-HDF-Exos induced a larger number of nodes (+46.2%, *p* < 0.0203) than did the same dose of SF-HaCaT-Exos, while the gains in the mean endothelial tube lengths were alike (*p* > 0.05) (Figure 7C–E).

Finally, a single dose (0.5 μg mL^−1^) of SF-HaCaT-Exos also increased the formation of HDMVECs’ endothelial tubes (12.7-fold, *p* < 0.0001) and nodes (4.2-fold, *p* < 0.0001) per microscopic field vs. untreated HDMVECs. On the other hand, the SF-HaCaT-Exos (0.5 μg mL^−1^) induced HDMVEC tubes of similar lengths (*p* < 0.05) but less numerous nodes per microscopic field (−39%, *p* < 0.0002) than did SF-HaCaT+HDF-Exos at the same dose (Figure 7C–E).

Altogether, the SF-HaCaT+HDF-Exos’ stimulatory effects on HDMVECs’ migration and tube/node formation proved that they were endowed with the highest proangiogenic power.

## 4. Discussion

In the past, various groups have used natural biopolymers, such as spiders’ or *Bombyx mori*’s SFs, as alternatives to chemically synthesized polymers to produce 3D scaffolds suitable for skin tissue engineering [16,17,18,19,20,124,125,126,127]. Various laboratories and our own have investigated skin equivalents composed of mulberry (from domesticated *Bombyx mori*) or non-mulberry (from wild silkworms, spiders, and more) SFs. Lee et al. [127] used biodegradable scaffolds of *Bombyx mori* SF electrospun nanofibers as artificial dermis substitutes, which induced Matriderm^®^-like wound healing rates while mitigating scar contraction. In turn, Sheikh et al. [128] modeled, via cold-plate electrospinning, a 3D SF nanofiber scaffold of high porosity and controlled thickness, thereby proposing it as an ideal candidate for skin reconstruction. Moreover, Park et al. [129] produced a novel type of SF-based 3D scaffold using electrospinning with added NaCl crystals, on which co-cultured keratinocytes and fibroblasts grew and differentiated. In addition, Hodgkinson et al. [124] showed that 3D poly(ethylene oxide) (PEO)/SF-based scaffolds composed of electrospun (250 nm in diameter) fibers supported the proliferation and the collagen types I and III synthesis of primary HDFs, while promoting the epidermal re-epithelization of ex vivo skin wound models. Moreover, covering wounds with 2D SF films accelerated skin regeneration while reducing collateral adverse events [125]. Finally, not only mulberry SF but also non-mulberry SFs accelerated skin wound healing [126].

Our laboratory first showed that, once grafted into the subcutaneous tissue of mice, FA-crosslinked 3D-SFnws guided, within 3 to 6 months, the de novo formation of a highly vascularized reticular connective tissue incorporating the SF microfibers but with no signs of a foreign body response, fibrosis, peripheral encapsulation, or hypertrophy of the superincumbent epidermis [21]. We also showed that long-term mixed (or direct) co-cultures of normal adult human keratinocytes and dermal fibroblasts could be successfully set up on these same FA-3D-SFnws, which therefore acted as dermo-epidermal equivalents [22]. However, at that time, we did not investigate any AGFs’ release. In view of the potential therapeutic applications suggested by our in vitro and in vivo results [21,23], we endeavored to produce an SF-based scaffold with improved mechanical characteristics. We succeeded in producing the C/H-3D-SFnws [36], whose mechanical features are similar to those of human skin [130]. Once implanted into mouse skin, the C/H-3D-SFnws guided the engineering of a richly vascularized reticular connective tissue, similarly to the FA-3D-SFnws. Altogether, these findings suggested that the contact between SF and mouse skin cells might induce the latter to release significant AGF amounts, perhaps conveyed by Exos, thereby driving the fast vascularization of the de-novo-engineered connective tissue [21,22,23]. Consistent with this hypothesis, our subsequent results showed that both HDFs and coronary artery SMCs, once stuck to 3D-SFnws, released AGF-enriched Exos endowed with strong angiogenic power [36,37]. In the present work, we produced the novel C/H-3D-SFnws/ESFN scaffold by adding a flat microporous layer of electrospun SF nanofibers to act as a basal membrane surrogate. To assess the biocompatibility of the new composite scaffolds, we set up indirect (i.e., separate yet intercommunicating) HaCaT+HDF co-cultures and monocultures of each cell type on it and verified their respective growth rates. Moreover, we collected the Exos from the two monocultures and the co-cultures on C/H-3D-SFnws/ESFN and from HaCaT monocultures on PS to assess the respective content of AGFs and their actual angiogenic power and, in parallel, to reveal the impact of the substrate (i.e., SF vs. PS) on them.

Our results showed that the indirect yet intercommunicating HaCaT+HDF co-cultures exhibited significantly higher growth rates than did the respective monocultures on the SF-based scaffold. It has been suggested that only direct contact between HDFs and keratinocytes co-cultured in vitro can enhance the proliferation of both cell types [131]. However, it is unlikely that direct intercellular contact played any crucial role in our system, as multiple lines of evidence indicate that even migrating cells cannot infiltrate ESFN-based structures [127,128,132]. Conversely, various reports have shown that bidirectional paracrine exchanges significantly promote the growth of both keratinocytes and fibroblasts and modulate the processes underlying the dynamic stages of skin wound healing [13,131,133,134,135]. It is likely that the mediators of such intercellular paracrine communications are sets of AGFs extracellularly released either in soluble form [27] or inside Exos [36,37] or other types of vesicles. The ESFN’s average pore diameters (Figure 1) are consistent with evidence [136] showing that pores of this size allow the bidirectional transfer of Exos through an ESFN-based structure, i.e., they allow reciprocal paracrine communications across the EFSN between the co-cultured HaCaTs and HDFs to take place, exerting beneficial effects on skin wounds [34,137,138,139,140].

Our present findings also show that the indirect co-cultures of HaCaTs and HDFs on C/H-3D-SFnws/ESFNs released Exos endowed with the highest cell growth-promoting and angiogenic power. In fact, such Exos conveyed 35 distinct AGFs, with 15 of these in remarkably increased amounts. HDFs or HaCaTs monocultured on the respective sides of the SF-based scaffold released Exos carrying smaller amounts of the same AGFs, hence having lower mitogenic and angiogenic effectiveness. However, as with the co-cultures, the monocultured cells may still have profited, in addition to the Exos’ autocrine effects, from their attachment to the combined nanoscale/microscale C/H-3D-SFnws/ESFN scaffold. In fact, albeit via mechanisms that remain mostly undetermined, patterned nanoscale/microscale structured scaffolds advance cells’ adhesion, growth, phenotypic expression, function, and migration [37,141,142]. In line with this, the beneficial effects exerted on wound healing by the SF-based dressings were driven by mostly not understood mechanisms in human cells. It is known that the biophysical properties of silk biomaterials (e.g., pore size and microstructure) can be used to control cellular processes, including cellular adhesion, proliferation, migration and differentiation, and ECM production (as reviewed in [143]). It has been suggested that the topography of fibrous scaffolds provides a unique microenvironment to modulate the paracrine function of MSCs [144], whose secretomes contain upregulated levels of anti-inflammatory and pro-angiogenic cytokines. In addition, the intrinsic biochemical signals due to the chemical properties of the SF may influence the cellular responses and function. Concerning rodents’ cells, Park et al. [142] used mouse NIH3T3 cells in a wound scratch assay and partial-thickness excision wounds in rats to show that the contact with SF activated the canonical NF-κB signaling pathway, which in turn increased the mRNA and protein expression of fibronectin, vimentin, cyclin D1, and VEGF. Thus, they concluded that the SF-contact-activated NF-κB signaling is crucial for wound healing. In turn, Liu T. et al. [145] reported that human fetal lung WI-38 fibroblasts cultured on regenerated SF films released VEGF, PDGF, FGF-2, and ANGPT-1. Consistent with our previous [21,22,23,36,37] and present work, the latter authors concluded that skin tissue engineering could be advanced by the use of pure-SF-made scaffolds. However, they did not investigate whether Exos played any role in this.

Our findings indicate that the contact with SF drives the activation not only of multiple but also (at least in part) similar signaling pathways in different human cell types.

In our earlier work [37], we showed that both the NF-κB signaling pathway and the TGF-β pathway were significantly activated in adult human coronary artery SMCs stuck to C/H-3D-SFnw scaffolds. This concurred with the release of Exos conveying increased amounts of 10 AGFs, which strongly stimulated in vitro HDVMECs’ angiogenic activity. Interestingly, in this work, we found that 8 out of 10 of the abovementioned AGFs were conveyed in significantly increased amounts by the Exos released from HaCaTs and/or HDFs mono- or co-cultured on C/H-3D-SFnws/ESFNs. In line with this, the AGFs total IIpv values in the double-antibody arrays challenged with the SF-HaCaT+HDF-Exos were significantly higher than those pertaining to membranes exposed to SF-HDF-Exos or SF-HaCaT-Exos (Figure 3). It should be borne in mind that the concurrent activities of all 35 Exos-transported AGF species brought about the promigratory and proangiogenic effects that we observed on the in vitro HDMVECs. These findings further strengthen the view that both the paracrine exchanges between the two cell types and the intracellular pathways activated by their attachment to the SF-based scaffolds significantly heightened the angiogenic power of the Exos that they released. In fact, HDFs or HaCaTs monocultured on PS discharged Exos transporting even smaller AGF amounts. Indeed, abundant lines of evidence [57,58,59,60,61,62,63,64,65,66,67,68,69,70,71,72,73,74,75,76,77,78,79,80,81,82,83,84,85,86,87,88,89,90,91,92,93,94,95,96,97,98,99,100,101,102,103,104,105,106,107,108,109,110,111,112,113,114,115,116,117,118,119,120,121,122,123] prove that the most represented of such Exo-conveyed AGFs could bring about a panoply of beneficial effects on the various skin constituents (see Table 1 for details).

Recently, the first groundbreaking multiomics analysis of the cellular processes and pathways of MSCs triggered by SF shed light on the great potential of the integrin/PI3K/Akt signaling pathway in regulating wound healing, skin repair/regeneration, and angiogenesis (as reviewed in [146]). It is known that insoluble SF (β-sheet form) induces integrin β1 expression in HUVECs and HDFs, which can promote cell migration, cell adhesion, and tissue repair under the guidance of microenvironment signals [147]. The Exos released in response to SF from the HaCaTs and or HDFs into the extracellular environment are indeed important paracrine signal carriers involved in the regulation of multiple cellular behaviors. They can enhance angiogenesis by promoting the expression and/or the direct transfer of pro-angiogenic factors into the recipient cells. These functions engage the interaction among the tetraspanins and ECM proteins expressed on the Exos’ surfaces with specific receptors (e.g., integrins) on recipient cells and promote Exo–cell docking. Moreover, there is also evidence that some Exos contain integrins cooperating in the docking of Exos onto certain cell types [148].

The roles played by the intracellular and/or extracellular microenvironments, the cell types involved, and other factors (e.g., various RNAs) affect the nature of the cargoes transported by Exos [149]. The molecular mechanisms invoked in various SF-attached human cell types constitute worthy objects of future basic and translational studies aimed at advancing the field of wound healing and of other human ailments. Therefore, the mechanisms regulating the specific qualitative and quantitative sorting of AGFs into the Exos produced by HaCaTs and/or HDFs mono- or co-cultured on C/H-3D-SFnws/ESFNs deserve further investigation.

A limitation of the present work is that we collected the Exos released during the first 15 days of HDFs and/or HaCaTs co-culturing or monoculturing on C/H-3D-SFnws/ESFNs. Therefore, our findings only relate to the Exo-mediated keratinocyte–fibroblast interactions occurring at the initial stages of wound healing.

## 5. Conclusions

Skin substitutes based on synthetic or natural polymers are attracting great interest as scaffolds for skin tissue engineering/regeneration. As previously discussed, the results so far reported present positive and negative aspects, as there are many advantages but also adverse effects of their use in the treatment of skin injuries. Our ambition is to overcome the current limitations and to contribute to solving the existing clinical problems by taking full advantage of the excellent characteristics of SF as a scaffolding material, as it offers high versatility in scaffold design. In this study, we realized a novel composite scaffold comprising an SF nonwoven component (C/H-3D-SFnw), with morphological and mechanical characteristics akin to those of human tissue (dermis), welded on one side to an electrospun layer (ESFN) as a surrogate for the basal lamina. The aim was to achieve spatial separation between the cell types involved in skin regeneration, while allowing the reciprocal exchange of growth factors and other bioactive agents. Besides responding to the requirements for an ideal scaffold for skin tissue regeneration, its composite structure combines the outstanding mechanical properties of native SF microfibers (C/H-3D-SFnw) and the enhanced biomimicking nature of the SF nanofibers (ESFN). The careful selection of starting materials and fine tuning of the fabrication technology and processing aids led to a composite structure where the two SF layers were perfectly integrated at the structural and functional levels and responded as a single body to external mechanical stresses. These properties are of chief importance in view of clinical translation, because manipulation and suturing during surgery is facilitated and the emergence of biomechanical mismatch at the implantation site is avoided. Therefore, our findings show that (*i*) the C/H-3D-SFnws/ESFN composite scaffold allows us to construct a novel type of skin equivalent/substitute; (*ii*) the contact of human keratinocytes and fibroblasts with SF nano- or microfibers drives their release of AGF surpluses via Exos; and (*iii*) by advancing the engineering of a vascularized subcutaneous tissue, dermis, and normal epidermis, these Exos effectively assist wound/burn and chronic ulcer healing. Moreover, our results show that the use of SF-based scaffolds to advance the optimal healing of skin wounds and chronic ulcers can provide huge benefits in clinical settings. As an example, the surpluses of AGFs released via Exos by SF-attached dermal and epidermal cells would particularly benefit diabetic patients, in whom angiogenesis is severely impaired [63,150,151]. Consistent with previous results [36,37], the present findings strengthen the view that properly structured SF-based skin equivalents possess superior characteristics as regards pliability, biocompatibility, nontoxicity, the mitigation of inflammation, the prevention of foreign body responses, hyperfibrosis prevention, a lack of immunogenicity, and the promotion of human cells’ growth, functioning, and exosomal release of AGFs. The latter feature would promote the vascularization and take of a grafted C/H-3D-SFnws/ESFN, as we observed in mice bearing less advanced types of SF-3D-nws [21,23] and as did Chou et al. in rats using β-sheet SF [147]. Therefore, our novel skin equivalent is worthy of additional study into its tissue engineering/regeneration-promoting properties and its translation into clinical and even veterinarian settings. Finally, the same skin equivalent/substitute model could be used to conduct skin-related pathophysiological, pharmacological, toxicological, and cosmetological investigations.

## Figures and Tables

**Table 1:** Actions of the significantly enriched AGFs conveyed by SF-HaCaT+HDF-Exos, SF-HaCaT-Exos, and SF-HDF-Exos on skin tissue components.

**AGF**	**Exos’ Sources**	**Growth- and Angiogenesis-Promoting Effects**
**Angiogenin**	HaCaT+HDF *	promotes the proliferation of basal epidermal keratinocytes, dermal fibroblasts, and hair follicle cells and increases the density of dermal collagen fibers and vessels [57,58,59]
**ANGTP-1**	HaCaT+HDF HDF **^#^**	controls blood vessel growth and permeability [60] regulates venous and lymphatic angiogenesis/remodeling in diabetic wounds/ulcers [61,62,63,64] promotes the reconstruction and final maturation of vascular networks [65,66] synergically with VEGF, enhances the development of a collateral circulation [67]
**ANGPT-2**	HDF	promotes ECs’ survival via the PI3K/Akt signaling pathway [68] stimulates angiogenesis via endothelial tube formation in mice [69]
**bFGF**	HaCaT+HDF HaCaT **^§^**	regulates both angiogenesis and arteriogenesis [70] enhances ECs’ and SMCs’ proliferation [71] regulates vascular remodeling and HDMVECs’ proliferation [72,73] potentiates the IL-1β-induced formation of HDMVECs’ tubes [74]
**CXCL5/ENA78**	HaCaT	shares the evolutionary proangiogenic ‘ELR’ motif with IL-8 and GRO-α [75] binds the CXC motif ligand receptor 2 (CXCR2), through the signaling of which chemokines mediate angiogenesis and wound healing [76]
**GRO-α/-β/-γ**	HaCaT+HDF HDF	strongly promotes ECs’ proliferation, migration, angiogenesis, and neovascularization, even in the absence of inflammation [77,78] GRO-α/CXCL1 shares the evolutionary proangiogenic ‘ELR’ motif with IL-8 and ENA78/CXCL5 [75] GRO-α/CXCL1 is upregulated in acute wounds, promotes keratinocytes’ migration and reepithelization [79,80] GRO-β/CXCL2 plays a key role in the angiogenesis-promoting breaking of junctions among ECs [81] HDF-released GRO-γ/CXCL3 partakes in HDMVECs’ tube formation in vitro [36]
**IL-1α**	HDF	promotes angiogenesis in vivo by inducing VEGF synthesis [82] activates the VEGF•VEGFR-2 signaling pathway [83] stimulates Platelet-Derived Growth Factor (PDGF) A chain [84] and bFGF expression [85] induces its own expression in vascular SMCs, exerting autocrine growth-stimulatory effects [86]
**IL-1β**	HaCaT+HDF	partially restores granulation tissue formation in wounds of NLRP-3 inflammasome-null mice during early wound healing [74] induces HDMVECs to form tubes and decreases the expression of von Willebrand factor and Platelet Endothelial Cell Adhesion Molecule (PECAM-1) synergically with bFGF [87]
**IL-4**	HDF	increases the expression of vascular cell adhesion molecule (VCAM)-1, IL-6, and MCP-1 [88] induces cytoskeletal rearrangements in both HUVECs and human coronary artery ECs [89] acts as a mild mitogen for both macro- and microvascular ECs [90,91,92]
**IL-6**	HaCaT+HDF	exerts autocrine growth-stimulating effects on vascular SMCs by inducing endogenous PDGF production [93] supports ECs’ proliferation and mobility [94]
**IL-8/CXCL8**	HaCaT+HDF HDF HaCaT	shares the evolutionary proangiogenic ‘ELR’ motif with GRO-α and ENA78/CXCL5 [75] strongly promotes ECs’ proliferation, migration, angiogenesis, and vascularization, even with no concurrent inflammation [77,78] mediates angiogenesis when over-released by human psoriatic keratinocytes [95] stimulates angiogenesis when secreted together with MCP-1/CCL2 by fibroblasts inside tumor metastases [96] cooperates with VEGF to recruit precursor ECs into skin keloids [97] plays a role in all phases of human wound healing [98] attracts neutrophils in the early stage of wound healing [99]
**MCP-1/CCL2**	HaCaT+HDF HaCaT	stimulates angiogenesis by cooperating with IL-8/CXCL8 [96] recruits monocytes, memory T cells, and dendritic cells, which also release growth factors and cytokines, to wounds or infected areas, promoting healing [100,101,102] normalizes neovascularization and collagen accumulation of skin wound healing in diabetic mice [103] partakes in mechano-transduction-induced hypertrophic scar formation [104]
**MMP-1**	HaCaT+HDF HDF	induces angiogenesis by activating protease-activated receptor-1 (PAR-1) [105]
**MMP-9**	HaCaT	aids wound closure by stimulating TGF-β collagen fiber retraction [106] removes the fibrinogen matrix promoting wound healing [107] stimulates HaCaTs’ proliferation and migration by cooperating with TIMP-1 [108]
**PDGF-BB**	HaCaT	advances re-epithelialization and dermal repair by granulation tissue, matrix synthesis and remodeling, angiogenesis, and wound contraction at sites of acute skin wounds [109,110]
**PIGF**	HaCaT+HDF HaCaT	partakes in the neovascularization of skin wound beds by small blood vessels [111] promotes vessel maturation by stimulating the proliferation and recruitment of SMCs and fibroblasts [111]
**TIE-2/** **ANGPT-1-R**	HaCaT+HDF HDF	binds ANGPT-1, ANGPT-2, ANGPT-3, and ANGPT-4 with alike affinity, activating tyrosine kinase signaling and downstream angiogenesis [61,62]
**TIMP-1**	HaCaT+HDF HDF HaCaT	blocks ECM’s breakdown by MMPs, thus stabilizing the basement membranes of wound beds, and promotes the HaCaTs’ proliferation and migration by cooperating with MMP-9 [112,113]
**TIMP-2**	HaCaT+HDF	blocks ECM’s breakdown by MMPs, thus stabilizing the basement membranes of wound beds, and inhibits MMP-2 activity, thus promoting skin wounds’ normal healing [112,113]
**uPAR**	HDF	as an exosomal surface protease, binds and degrades ECM proteins such as collagens, laminin, and fibronectin, promoting neoangiogenesis and wound repair [114,115] advances angiogenesis by quelling the expression of its crucial negative regulator, i.e., the phosphatase and tensin homolog (PTEN) [116] once complexed with vitronectin, advances ECs’ adhesion and migration [117] in soluble full-length form, accelerates wound healing via skin epidermal cells and possibly peri-wound dermal fibroblasts [118]
**VEGF-A**	HaCaT+HDF	powerfully stimulates HDMVECs’ mitogenesis [119] aids wound healing in diabetes once released from keratinocytes [120] induces angiogenesis once overexpressed and secreted by human psoriatic keratinocytes in vitro [121] stimulates keratinocytes’ migration and myofibroblasts’ transition once oversecreted by human senescent fibroblasts [122]
**VEGF-D**	HaCaT+HDF	crucially regulates angiogenesis, lymphangiogenesis, and ECs’ growth and stimulates myofibroblast proliferation, migration, and collagen synthesis [123] drives myofibroblasts’ transition and keratinocytes’ migration once over-secreted by human senescent fibroblasts [122]

* HaCaT+HDF, increased content in SF-HaCaT+HDF-Exos compared to the content of SF-HaCaT-Exos and SF-HDF-Exos. **^#^** HaCaT, heightened content in SF-HaCaT-Exos as compared to the content of PS-HaCaT-Exos. **^§^** HDF, increased content in SF-HDF-Exos as compared to the content of PS-HDF-Exos.

## Data Availability

The datasets of this study are available upon request to the corresponding author.

## Supplementary Materials

The following supporting information can be downloaded at: https://www.mdpi.com/article/10.3390/cells12141827/s1, Figure S1: The double-antibody array membranes showing the AGFs carried by the exosomes released from monocultured or co-cultured HDFs and HaCaTs grown either on C/H-3D-SFnws/ESFN scaffolds or PS.

## Abbreviations

*2D*	Two-Dimensional
*3D*	Three-Dimensional
*3D-SFnws*	Three-Dimensional Silk Fibroin Nonwoven(s)
*ANGPT-*	Angiopoietin-.
*AGF(s)*	Angiogenic/Growth Factor(s)
*ATR-FTIR*	Attenuated Total Reflectance Fourier Transform Infrared (Spectroscopy)
*bFGF*	Basic Fibroblast Growth Factor
*CCL*	Chemokine (C-C Motif) Ligand
*C/H-3D-SFnws*	Carded/Hydroentangled Three-Dimensional SF Microfiber-Based Nonwoven (Dermis Scaffold)
*CXCL-*	C-X-C Motif Chemokine Ligand-
*DiIC18(3)*	1,1′-Dioctadecyl-3,3,3′,3′-Tetramethyl-Indocarbocyanine Perchlorate
*DMEM*	Dulbecco-Modified Eagle’s Minimum Essential Medium
*DSC*	Differential Scanning Calorimetry
*ECBM*	Endothelial Cell Basal Medium
*ECGM*	Endothelial Cell Growth Medium
*ECM*	Extracellular Matrix
*ECs*	Endothelial Cells
*ELISA*	Enzyme-Linked Immuno-Sorbent Assay
*EMIMAc*	1-Ethyl-3-Methylimidazolium Acetate
*ESFN(s)*	Electrospun Two-Dimensional SF Nanofiber(S)-Based Layer (Basal Lamina Surrogate)
*Exo/s*	Exosome/s
*FA*	Formic Acid
*GRO-*	Growth-Regulated Oncogene-
*HaCaT*	Spontaneously Transformed Human Keratinocyte Cell Line
*HDF*	Human Dermal Fibroblasts
*HDMVECs*	Human Dermal Microvascular Endothelial Cells
*IIpv*	Integrated Intensity Pixel Volume (Value)
*IL-*	Interleukin-
*MVB*	Multi-Vesicular Body
*MCP-*	Monocyte Chemoattractant Protein-
*MMP-*	Matrix Metallo-Peptidase-
*MSC-*	Mesenchymal Stem Cell
*NF-kB*	Nuclear Factor k Light Chain Enhancer of Activated B Cells
*PIGF*	Placental Growth Factor
*PS*	Polystyrene
*PS-HaCaT-Exos*	Exosomes Released from HaCaT Monocultures on PS
*PS-HDF-Exos*	Exosomes Released from HDF Monocultures on PS
*SEM*	Scanning Electron Microscopy.
*SF-HaCaT-Exos*	Exosomes Released from HaCaT Monocultures on C/H-3D-SFnws/ESFN
*SF-HaCaT+HDF-Exos*	Exosomes Released from HaCaT+HDF Co-Cultures on C/H-3D-SFnws/ESFN
*SF-HDF-Exos*	Exosomes Released from HDF Monocultures on C/H-3D-SFnws/ESFN
*SMCs*	Smooth Muscle Cells
*TGF-*	Transforming Growth Factor-
*TIE-2*	Angiopoietin-1 Receptor
*TIMP-*	Tissue Inhibitor of Metallo-Peptidase-
*uPAR*	Urokinase-Type Plasminogen Activator Receptor
*VEGF-*	Vascular Endothelial Growth Factor-

## References

[1] Nussbaum S.R., Carter M.J., Fife C.E., DaVanzo J., Haught R., Nusgart M., Cartwright D. (2018). An Economic Evaluation of the Impact, Cost, and Medicare Policy Implications of Chronic Nonhealing Wounds. Value Health.

[2] Sen C.K. (2021). Human Wound and Its Burden: Updated 2020 Compendium of Estimates. Adv. Wound Care.

[3] Velnar T., Bailey T., Smrkolj V. (2009). The wound healing process: An overview of the cellular and molecular mechanisms. J. Int. Med. Res..

[4] Han G., Ceilley R. (2017). Chronic Wound Healing: A Review of Current Management and Treatments. Adv. Ther..

[5] Vig K., Chaudhari A., Tripathi S., Dixit S., Sahu R., Pillai S., Dennis V.A., Singh S.R. (2017). Advances in Skin Regeneration Using Tissue Engineering. Int. J. Mol. Sci..

[6] Chiarini A., Dal Prà I., Armato U. (2007). In vitro and in vivo characteristics of frozen/thawed neonatal pig split-skin strips: A novel biologically active dressing for areas of severe, acute or chronic skin loss. Int. J. Mol. Med..

[7] O’Connor N.E., Mulliken J., Banks-Schlegel S., Green H. (1981). Grafting of burns with cultured epithelium prepared from autologous epidermal cells. Lancet.

[8] Wang Y., Armato U., Wu J. (2020). Targeting Tunable Physical Properties of Materials for Chronic Wound Care. Front. Bioeng. Biotechnol..

[9] MacNeil S. (2007). Progress and opportunities for tissue-engineered skin. Nature.

[10] Metcalfe A.D., Ferguson M.W. (2007). Tissue engineering of replacement skin: The crossroads of biomaterials, wound healing, em-bryonic development, stem cells and regeneration. J. R. Soc. Interface.

[11] Gurtner G.C., Chapman M.A. (2016). Regenerative Medicine: Charting a New Course in Wound Healing. Adv. Wound Care.

[12] Place E.S., George J.H., Williams C.K., Stevens M.M. (2009). Synthetic polymer scaffolds for tissue engineering. Chem. Soc. Rev..

[13] McMillan J.R., Akiyama M., Tanaka M., Yamamoto S., Goto M., Abe R., Sawamura D., Shimomura M., Shimizu H. (2007). Small-diameter porous poly (epsilon-caprolactone) films enhance adhesion and growth of human cultured epidermal keratinocyte and dermal fibroblast cells. Tissue Eng..

[14] Farokhi M., Mottaghitalab F., Fatahi Y., Khademhosseini A., Kaplan D.L. (2018). Overview of Silk Fibroin Use in Wound Dressings. Trends Biotechnol..

[15] Sutherland T.D., Young J.H., Weisman S., Hayashi C.Y., Merritt D.J. (2010). Insect silk: One name, many materials. Annu. Rev. Entomol..

[16] Naskar D., Barua R.R., Ghosh A.K., Kundu S.C., Kundu S.C. (2014). Introduction to silk biomaterials. Silk Biomaterials for Tissue Engineering and Regenerative Medicine.

[17] Valluzzi R., Gido S.P., Muller W., Kaplan D.L. (1999). Orientation of silk III at the air-water interface. Int. J. Biol. Macromol..

[18] Kundu B., Rajkhowa R., Kundu S.C., Wang X. (2013). Silk fibroin biomaterials for tissue regenerations. Adv. Drug Deliv. Rev..

[19] Bucciarelli A., Motta A. (2022). Use of Bombyx mori silk fibroin in tissue engineering: From cocoons to medical devices, challenges, and future perspectives. Biomater. Adv..

[20] Armato U., Dal Prà I., Chiarini A., Freddi G. (2011). Will silk fibroin nanofiber scaffolds ever hold a useful place in Translational Regenerative Medicine?. Int. J. Burns Trauma.

[21] Dal Prà I., Freddi G., Minic J., Chiarini A., Armato U. (2005). De novo engineering of reticular connective tissue in vivo by silk fibroin nonwoven materials. Biomaterials.

[22] Dal Prà I., Chiarini A., Boschi A., Freddi G., Armato U. (2006). Novel dermo-epidermal equivalents on silk fibroin-based formic acid- crosslinked three-dimensional nonwoven devices with prospective applications in human tissue engineering/regeneration/repair. Int. J. Mol. Med..

[23] Chiarini A., Freddi G., Liu D., Armato U., Dal Prà I. (2016). Biocompatible silk noil-based three-dimensional carded-needled nonwoven scaffolds guide the engineering of a novel skin connective tissue. Tissue Eng. Part A.

[24] Booth A.M., Fang Y., Fallon J.K., Yang J.M., Hildreth J.E., Gould S.J. (2006). Exosomes and HIV gag bud from endosome-like domains of the T cell plasma membrane. J. Cell Biol..

[25] Keller S., Sanderson M.P., Stoeck A., Altevogt P. (2006). Exosomes: From biogenesis and secretion to biological function. Immunol. Lett..

[26] Than U.T.T., Guanzon D., Leavesley D., Parker T. (2017). Association of Extracellular Membrane Vesicles with Cutaneous Wound Healing. Int. J. Mol. Sci..

[27] van der Pol E., Böing A.N., Harrison P., Sturk A., Nieuwland R. (2012). Classification, functions, and clinical relevance of extracellular vesicles. Pharmacol. Rev..

[28] Gartz M., Strande J.L. (2018). Examining the Paracrine Effects of Exosomes in Cardiovascular Disease and Repair. J. Am. Heart Assoc..

[29] Hood J.L., Pan H., Lanza G.M., Wickline S.A. (2009). Consortium for Translational Research in Advanced Imaging and Nanomedicine (C-TRAIN). Paracrine induction of endothelium by tumor exosomes. Lab. Investig..

[30] Sheller-Miller S., Trivedi J., Yellon S.M., Menon R. (2019). Exosomes Cause Preterm Birth in Mice: Evidence for Paracrine Signaling in Pregnancy. Sci. Rep..

[31] Lai R.C., Arslan F., Lee M.M., Sze N.S., Choo A., Chen T.S., Salto-Tellez M., Timmers L., Lee C.N., El Oakley R.M. (2010). Exosome secreted by MSC reduces myocardial ischemia/reperfusion injury. Stem Cell Res..

[32] Hu P., Yang Q., Wang Q., Shi C., Wang D., Armato U., Dal Prà I., Chiarini A. (2019). Mesenchymal stromal cells-exosomes: A promising cell-free therapeutic tool for wound healing and cutaneous regeneration. Burns Trauma.

[33] Golchin A., Hosseinzadeh S., Ardeshirylajimi A. (2018). The exosomes released from different cell types and their effects in wound healing. J. Cell. Biochem..

[34] Zhou Y., Zhao B., Zhang X.L., Lu Y.J., Lu S.T., Cheng J., Fu Y., Lin L., Zhang N.Y., Li P.X. (2021). Combined topical and systemic administration with human adipose-derived mesenchymal stem cells (hADSC) and hADSC-derived exosomes markedly promoted cutaneous wound healing and regeneration. Stem Cell Res. Ther..

[35] An Y., Lin S., Tan X., Zhu S., Nie F., Zhen Y., Gu L., Zhang C., Wang B., Wei W. (2021). Exosomes from adipose-derived stem cells and application to skin wound healing. Cell Prolif..

[36] Hu P., Chiarini A., Wu J., Freddi G., Nie K., Armato U., Dal Prà I. (2021). Exosomes of adult human fibroblasts cultured on 3D silk fibroin nonwovens intensely stimulate neoangiogenesis. Burns Trauma.

[37] Hu P., Chiarini A., Wu J., Wei Z., Armato U., Dal Prà I. (2022). Adult Human Vascular Smooth Muscle Cells on 3D Silk Fibroin Nonwovens Release Exosomes Enriched in Angiogenic and Growth-Promoting Factors. Polymers.

[38] Altman G.H., Diaz F., Jakuba C., Calabro T., Horan R.L., Chen J., Lu H., Richmond J., Kaplan D.L. (2003). Silk-based biomaterials. Biomaterials.

[39] Rockwood D.N., Preda R.C., Yücel T., Wang X., Lovett M.L., Kaplan D.L. (2011). Materials fabrication from Bombyx mori silk fibroin. Nat. Protoc..

[40] Marelli B., Alessandrino A., Farè S., Freddi G., Mantovani D., Tanzi M.C. (2010). Compliant electrospun silk fibroin tubes for small vessel bypass grafting. Acta Biomater..

[41] Alessandrino A. (2016). Process for the Production of a Composite Structure Consisting of Coupled Silk Fibroin Microfibers and Nanofibers, Composite Structure Thus Obtained and Its Use as Implantable Medical Device. WO.

[42] Boukamp P., Petrussevska R.T., Breitkreutz D., Hornung J., Markham A., Fusenig N.E. (1988). Normal keratinization in a spontaneously Immortalized Aneuploid Human Keratinocyte Cell Line. J. Cell Biol..

[43] Schoop V.M., Mirancea N., Fusenig N.E. (1999). Epidermal organization and differentiation of HaCaT keratinocytes in organotypic co-culture with human dermal fibroblasts. J. Investig. Dermatol..

[44] Kehe K., Abend M., Kehe K., Ridi R., Peter R.U., van Beuningen D. (1999). Tissue engineering with HaCaT cells and a fibroblast cell line. Arch. Dermatol. Res..

[45] Chiarini A., Armato U., Gardenal E., Gui L., Dal Prà I. (2017). Amyloid β-exposed human astrocytes overproduce Phospho-tau and overrelease it within exosomes, effects suppressed by Calcilytic NPS 2143-further implications for Alzheimer’s therapy. Front. Neurosci..

[46] Nakamura K., Jinnin M., Harada M., Kudo H., Nakayama W., Inoue K., Ogata A., Kajihara I., Fukushima S., Ihn H. (2016). Altered expression of CD63 and exosomes in scleroderma dermal fibroblasts. J. Dermatol. Sci..

[47] Kowal J., Tkach M., Théry C. (2014). Biogenesis and secretion of exosomes. Curr. Opin. Cell Biol..

[48] Ventress J.K., Partridge L.J., Read R.C., Cozens D., MacNeil S., Monk P.N. (2016). Peptides from Tetraspanin CD9 Are Potent Inhibitors of Staphylococcus Aureus Adherence to Keratinocytes. PLoS ONE.

[49] Armato U., Romano F., Andreis P.G., Paccagnella L., Marchesini C. (1986). Growth stimulation and apoptosis induced in cultures of neonatal rat liver cells by repeated exposures to epidermal growth factor/urogastrone with or without associated pancreatic hormones. Cell Tissue Res..

[50] Zhang K., Mo X., Huang C., He C., Wang H. (2010). Electrospun scaffolds from silk fibroin and their cellular compatibility. J. Biomed. Mater. Res. A.

[51] Lotz B., Colonna Cesari F. (1979). The chemical structure and the crystalline structures of Bombyx mori silk fibroin. Biochimie.

[52] Biagiotti M., Bassani G.A., Chiarini A., Vincoli V.T., Dal Prà I., Cosentino C., Alessandrino A., Taddei P., Freddi G. (2022). Electrospun Silk Fibroin Scaffolds for Tissue Regeneration: Chemical, Structural, and Toxicological Implications of the Formic Acid-Silk Fibroin Interaction. Front. Bioeng. Biotechnol..

[53] Lemaître G., Lamartine J., Pitaval A., Vaigot P., Garin J., Bouet S., Petat C., Soularue P., Gidrol X., Martin M.T. (2004). Expression profiling of genes and proteins in HaCaT keratinocytes: Proliferating versus differentiated state. J. Cell. Biochem..

[54] Gailit J., Clark R.A. (1994). Wound repair in the context of extracellular matrix. Curr. Opin. Cell Biol..

[55] Angel P., Szabowski A. (2002). Function of AP-1 target genes in mesenchymal-epithelial cross-talk in skin. Biochem. Pharmacol..

[56] Maas-Szabowski N., Shimotoyodome A., Fusenig N.E. (1999). Keratinocyte growth regulation in fibroblast co-cultures via a double paracrine mechanism. J. Cell Sci..

[57] Zhou N., Fan W., Li M. (2009). Angiogenin is expressed in human dermal papilla cells and stimulates hair growth. Arch. Dermatol. Res..

[58] Yurina N.V., Ageeva T.A., Goryachkin A.M., Varaksin N.A., Ryabicheva T.G., Ostanin A.A., Chernykh E.R., Romashchenko A.V., Proskurina A.S., Bogachev S. (2021). Effects of Recombinant Angiogenin on Collagen Fiber Formation and Angiogenesis in the Dermis of Wistar Rats. Clin. Cosmet. Investig. Dermatol..

[59] Sadagopan S., Sharma-Walia N., Veettil M.V., Bottero V., Levine R., Vart R.J., Chandran B. (2009). Kaposi’s sarcoma-associated herpesvirus upregulates angiogenin during infection of human dermal microvascular endothelial cells, which induces 45S rRNA synthesis, antiapoptosis, cell proliferation, migration, and angiogenesis. J. Virol..

[60] Thurston G. (2002). Complementary actions of VEGF and angiopoietin-1 on blood vessel growth and leakage. J. Anat..

[61] Augustin H.G., Koh G.-Y. (2017). Organotypic vasculature: From descriptive heterogeneity to functional pathophysiology. Science.

[62] Saharinen P., Leppänen V.M., Alitalo K. (2017). Snapshot: Angiopoietins and their functions. Cell.

[63] Balaji S., Han N., Moles C., Shaaban A.F., Bollyky P.L., Crombleholme T.M., Keswani S.G. (2015). Angiopoietin-1 improves endothelial progenitor cell-dependent neovascularization in diabetic wounds. Surgery.

[64] Kim I., Kim H.G., Moon S.O., Chae S.W., So J.N., Koh K.N., Ahn B.C., Koh G.Y. (2000). Angiopoietin-1 induces endothelial cell sprouting through the activation of focal adhesion kinase and plasmin secretion. Circ. Res..

[65] Kim I., Kim H.G., So J.N., Kim J.H., Kwak H.J., Koh G.Y. (2000). Angiopoietin-1 regulates endothelial cell survival through the phosphatidylinositol 3′-Kinase/Akt signal transduction pathway. Circ. Res..

[66] Yancopoulos G.D., Davis S., Gale N.W., Rudge J.S., Wiegand S.J., Holash J. (2000). Vascular-specific growth factors and blood vessel formation. Nature.

[67] Chae J.K., Kim I., Lim S.T., Chung M.J., Kim W.H., Kim H.G., Ko J.K., Koh G.Y. (2000). Coadministration of angiopoietin-1 and vascular endothelial growth factor enhances collateral vascularization. Arterioscler. Thromb. Vasc. Biol..

[68] Kim I., Kim J.H., Moon S.O., Kwak H.J., Kim N.G., Koh G.Y. (2000). Angiopoietin-2 at high concentration can enhance endothelial cell survival through the phosphatidylinositol 3′-kinase/Akt signal transduction pathway. Oncogene.

[69] Gu J., Zhang Y., Han Z., Gao L., Cui J., Sun Y., Niu Y., You B., Huang C.P., Chang C. (2020). Targeting the ERβ/Angiopoietin-2/Tie-2 signaling-mediated angiogenesis with the FDA-approved anti-estrogen Faslodex to increase the Sunitinib sensitivity in RCC. Cell Death Dis..

[70] Presta M., Dell’Era P., Mitola S., Moroni E., Ronca R., Rusnati M. (2005). Fibroblast growth factor/fibroblast growth factor receptor system in angiogenesis. Cytokine Growth Factor Rev..

[71] Tomanek R.J., Hansen H.K., Christensen L.P. (2008). Temporally expressed PDGF and FGF-2 regulate embryonic coronary artery formation and growth. Arterioscler. Thromb. Vasc. Biol..

[72] Holnthoner W., Pillinger M., Groger M., Wolff K., Ashton A.W., Albanese C., Neumeister P., Pestell R.G., Petzelbauer P. (2002). Fibroblast growth factor-2 induces Lef/Tcf-dependent transcription in human endothelial cells. J. Biol. Chem..

[73] Wang X., Xiao Y., Mou Y., Zhao Y., Blankesteijn W.M., Hall J.L. (2002). A role for the beta-catenin/T-cell factor signaling cascade in vascular remodeling. Circ. Res..

[74] Romero L.I., Zhang D.N., Herron G.S., Karasek M.A. (1997). Interleukin-1 induces major phenotypic changes in human skin microvascular endothelial cells. J. Cell. Physiol..

[75] Pappa C.A., Tsirakis G., Kanellou P., Kaparou M., Stratinaki M., Xekalou A., Alegakis A., Boula A., Stathopoulos E.N., Alexandrakis M.G. (2011). Monitoring serum levels ELR+ CXC chemokines and the relationship between microvessel density and angiogenic growth factors in multiple myeloma. Cytokine.

[76] Maarof M., Law J.X., Chowdhury S.R., Khairoji K.A., Saim A.B., Idrus R.B. (2016). Secretion of wound healing mediators by single and bi-layer skin substitutes. Cytotechnology.

[77] Lan C.C., Wu C.S., Huang S.M., Wu I.H., Chen G.S. (2013). High-glucose environment enhanced oxidative stress and increased interleukin-8 secretion from keratinocytes: New insights into impaired diabetic wound healing. Diabetes.

[78] Strieter R.M., Polverini P.J., Kunkel S.L., Arenberg D.A., Burdick M.D., Kasper J., Dzuiba J., Van Damme J., Walz A., Marriott D. (1995). The functional role of the ELR motif in CXC chemokine-mediated angiogenesis. J. Biol. Chem..

[79] Engelhardt E., Toksoy A., Goebeler M., Debus S., Bröcker E.B., Gillitzer R. (1998). Chemokines IL-8, GRO alpha, MCP-1, IP-10, and Mig are sequentially and differentially expressed during phase specific infiltration of leukocyte subsets in human wound healing. Am. J. Pathol..

[80] Christopherson K., Hromas R. (2001). Chemokine regulation of normal and pathologic immune responses. Stem Cells.

[81] Girbl T., Lenn T., Perez L., Rolas L., Barkaway A., Thiriot A., Del Fresno C., Lynam E., Hub E., Thelen M. (2018). Distinct compartmentalization of the chemokines CXCL1 and CXCL2 and the atypical receptor ACKR1 determine discrete stages of neutrophil diapedesis. Immunity.

[82] Fahey E., Doyle S.L. (2019). IL-1 Family Cytokine Regulation of Vascular Permeability and Angiogenesis. Front. Immunol..

[83] Salven P., Hattori K., Heissig B., Rafii S. (2002). Interleukin-1alpha promotes angiogenesis in vivo via VEGFR-2 pathway by inducing inflammatory cell VEGF synthesis and secretion. FASEB J..

[84] Raines E.W., Dower S.K., Ross R. (1989). Interleukin-1 mitogenic activity for fibroblasts and smooth muscle cells is due to PDGF-AA. Science.

[85] Gay C.G., Winkles J.A. (1991). Interleukin 1 regulates heparin-binding growth factor 2 gene expression in vascular smooth muscle cells. Proc. Natl. Acad. Sci. USA.

[86] Beasley D., Cooper A.L. (1999). Constitutive expression of interleukin-1α precursor promotes human vascular smooth muscle cell proliferation. Am. J. Physiol. Heart Circ. Physiol..

[87] Weinheimer-Haus E.M., Mirza R.E., Koh T.J. (2015). Nod-like receptor protein-3 inflammasome plays an important role during early stages of wound healing. PLoS ONE.

[88] Lee Y.W., Eum S.Y., Chen K.C., Hennig B., Toborek M. (2004). Gene expression profile in interleukin-4-stimulated human vascular endothelial cells. Mol. Med..

[89] Skaria T., Burgener J., Bachli E., Schoedon G. (2016). IL-4 Causes Hyperpermeability of Vascular Endothelial Cells through Wnt5A Signaling. PLoS ONE.

[90] Klein N.J., Rigley K.P., Callard R.E. (1993). IL-4 regulates the morphology, cytoskeleton, and proliferation of human umbilical vein endothelial cells: Relationship between vimentin and CD23. Int. Immunol..

[91] Toi M., Harris A.L., Bicknell R. (1991). Interleukin-4 is a potent mitogen for capillary endothelium. Biochem. Biophys. Res. Commun..

[92] Fukushi J., Morisaki T., Shono T., Nishie A., Torisu H., Ono M., Kuwano M. (1998). Novel biological functions of interleukin-4: Formation of tube-like structures by vascular endothelial cells in vitro and angiogenesis in vivo. Biochem. Biophys. Res. Commun..

[93] Ikeda U., Ikeda M., Oohara T., Oguchi A., Kamitani T., Tsuruya Y., Kano S. (1991). Interleukin 6 stimulates growth of vascular smooth muscle cells in a PDGF-dependent manner. Am. J. Physiol..

[94] Ljungberg L.U., Zegeye M.M., Kardeby C., Fälker K., Repsilber D., Sirsjö A. (2020). Global Transcriptional Profiling Reveals Novel Autocrine Functions of Interleukin 6 in Human Vascular Endothelial Cells. Mediat. Inflamm..

[95] Nickoloff B.J., Mitra R.S., Varani J., Dixit V.M., Polverini P.J. (1994). Aberrant production of interleukin-8 and thrombospondin-1 by psoriatic keratinocytes mediates angiogenesis. Am. J. Pathol..

[96] Pausch T.M., Aue E., Wirsik N.M., Valls A.F., Shen Y., Radhakrishnan P., Hackert T., Schneider M., Schmidt T. (2020). Metastasis-associated fibroblasts promote angiogenesis in metastasized pancreatic cancer via the CXCL8 and the CCL2 axes. Sci. Rep..

[97] Tanaka R., Umeyama Y., Hagiwara H., Ito-Hirano R., Fujimura S., Mizuno H., Ogawa R. (2019). Keloid patients have higher peripheral blood endothelial progenitor cell counts and CD34^+^ cells with normal vasculogenic and angiogenic function that overexpress vascular endothelial growth factor and interleukin-8. Int. J. Dermatol..

[98] Rennekampff H.O., Hansbrough J.F., Kiessig V., Doré C., Sticherling M., Schröder J.M. (2000). Bioactive interleukin-8 is expressed in wounds and enhances wound healing. J. Surg. Res..

[99] Keeley E.C., Mehrad B., Strieter R.M. (2008). Chemokines as mediators of neovascularization. Arterioscler. Thromb. Vasc. Biol..

[100] Carr M.W., Roth S.J., Luther E., Rose S.S., Springer T.A. (1994). Monocyte chemoattractant protein 1 acts as a T-lymphocyte chemoattractant. Proc. Natl. Acad. Sci. USA.

[101] Xu L.L., Warren M.K., Rose W.L., Gong W., Wang J.M. (1996). Human recombinant monocyte chemotactic protein and other C-C chemokines bind and induce directional migration of dendritic cells in vitro. J. Leukoc. Biol..

[102] Ridiandries A., Tan J., Bursill C.A. (2018). The Role of Chemokines in Wound Healing. Int. J. Mol. Sci..

[103] Ishida Y., Kuninaka Y., Nosaka M., Furuta M., Kimura A., Taruya A., Yamamoto H., Shimada E., Akiyama M., Mukaida N. (2019). CCL2-Mediated Reversal of Impaired Skin Wound Healing in Diabetic Mice by Normalization of Neovascularization and Collagen Accumulation. J. Investig. Dermatol..

[104] Menten P., Wuyts A., Van Damme J. (2002). Macrophage inflammatory protein-1. Cytokine Growth Factor Rev..

[105] Fan H.X., Chen Y., Ni B.X., Wang S., Sun M., Chen D., Zheng J.H. (2015). Expression of MMP-1/PAR-1 and patterns of invasion in oral squamous cell carcinoma as potential prognostic markers. OncoTargets Ther..

[106] Kobayashi T., Kim H., Liu X., Sugiura H., Kohyama T., Fang Q., Wen F.Q., Abe S., Wang X., Atkinson J.J. (2014). Matrix metalloproteinase-9 activates TGF-β and stimulates fibroblast contraction of collagen gels. Am. J. Physiol. Lung Cell. Mol. Physiol..

[107] Mohan R., Chintala S.K., Jung J.C., Villar W.V., McCabe F., Russo L.A., Lee Y., McCarthy B.E., Wollenberg K.R., Jester J.V. (2002). Matrix metalloproteinase gelatinase B (MMP-9) coordinates and effects epithelial regeneration. J. Biol. Chem..

[108] Yang C., Luo L., Bai X., Shen K., Liu K., Wang J., Hu D. (2020). Highly-expressed microRNA-21 in adipose derived stem cell exosomes can enhance the migration and proliferation of the HaCaT cells by increasing the MMP-9 expression through the PI3K/AKT pathway. Arch. Biochem. Biophys..

[109] Ansel J.C., Tiesman J.P., Olerud J.E., Krueger J.G., Krane J.F., Tara D.C., Shipley G.D., Gilbertson D., Usui M.L., Hart C.E. (1993). Human keratinocytes are a major source of cutaneous platelet-derived growth factor. J. Clin. Investig..

[110] Md Fadilah N.I., Mohd Abdul Kader Jailani M.S., Badrul Hisham M., Sunthar Raj N., Shamsuddin S.A., Ng M.H., Fauzi M.B., Maarof M. (2022). Cell secretomes for wound healing and tissue regeneration: Next generation acellular based tissue engineered products. J. Tissue Eng..

[111] De Falco S. (2012). The discovery of placenta growth factor and its biological activity. Exp. Mol. Med..

[112] Bourboulia D., Stetler-Stevenson W.G. (2010). Matrix metalloproteinases (MMPs) and tissue inhibitors of metalloproteinases (TIMPs): Positive and negative regulators in tumor cell adhesion. Semin. Cancer Biol..

[113] Vaalamo M., Leivo T., Saarialho-Kere U. (1999). Differential expression of tissue inhibitors of metalloproteinases (TIMP-1, -2, -3, and -4) in normal and aberrant wound healing. Hum. Pathol..

[114] Mu W., Rana S., Zöller M. (2013). Host matrix modulation by tumor exosomes promotes motility and invasiveness. Neoplasia.

[115] Su S.-C., Lin C.-W., Yang W.-E., Fan W.-L., Yang S.-F. (2016). The urokinase-type plasminogen activator (uPA) system as a biomarker and therapeutic target in human malignancies. Expert Opin. Ther. Targets.

[116] Unseld M., Chilla A., Pausz C., Mawas R., Breuss J., Zielinski C., Schabbauer G., Prager G.W. (2015). PTEN expression in endothelial cells is down-regulated by uPAR to promote angiogenesis. Thromb. Haemost..

[117] Preissner K.T., Reuning U. (2011). Vitronectin in vascular context: Facets of a multitalented matricellular protein. Semin. Thromb. Hemost..

[118] Cooper F., Overmiller A.M., Loder A., Brennan-Crispi D.M., McGuinn K.P., Marous M.R., Freeman T.A., Riobo-Del Galdo N.A., Siracusa L.D., Wahl J.K. (2018). Enhancement of cutaneous wound healing by Dsg2 augmentation of uPAR secretion. J. Investig. Dermatol..

[119] Detmar M., Yeo K.T., Nagy J.A., Van de Water L., Brown L.F., Berse B., Elicker B.M., Ledbetter S., Dvorak H.F. (1995). Keratinocyte-derived vascular permeability factor (vascular endothelial growth factor) is a potent mitogen for dermal microvascular endothelial cells. J. Investig. Dermatol..

[120] Wang Y., Graves D.T. (2020). Keratinocyte Function in Normal and Diabetic Wounds and Modulation by FOXO1. J. Diabetes Res..

[121] Chiarini A., Dal Pra I., Pacchiana R., Menapace L., Zumiani G., Zanoni M., Armato U. (2006). Comano’s (Trentino) thermal water interferes with the expression and secretion of vascular endothelial growth factor-A protein isoforms by cultured human psoriatic keratinocytes: A potential mechanism of its anti-psoriatic action. Int. J. Mol. Med..

[122] Hou J., Kim S. (2018). Possible role of ginsenoside Rb1 in skin wound healing via regulating senescent skin dermal fibroblast. Biochem. Biophys. Res. Commun..

[123] Zhao T., Zhao W., Meng W., Liu C., Chen Y., Bhattacharya S.K., Sun Y. (2016). Vascular endothelial growth factor-D mediates fibrogenic response in myofibroblasts. Mol. Cell. Biochem..

[124] Hodgkinson T., Yuan X.F. (2014). Electrospun silk fibroin fiber diameter influences in vitro dermal fibroblast behavior and promotes healing of ex vivo wound models. J. Tissue Eng..

[125] Zhang W., Chen L., Chen J., Wang L., Gui X., Ran J., Xu G., Zhao H., Zeng M., Ji J. (2017). Silk Fibroin Biomaterial Shows Safe and Effective Wound Healing in Animal Models and a Randomized Controlled Clinical Trial. Adv. Healthc. Mater..

[126] Chouhan D., Chakraborty B., Nandi S.K., Mandal B.B. (2017). Role of non-mulberry silk fibroin in deposition and regulation of extracellular matrix towards accelerated wound healing. Acta Biomater..

[127] Lee O.J., Ju H.W., Kim J.H., Lee J.M., Ki C.S., Kim J.H., Moon B.M., Park H.J., Sheikh F.A., Park C.H. (2014). Development of artificial dermis using 3D electrospun silk fibroin nanofiber matrix. J. Biomed. Nanotechnol..

[128] Sheikh F.A., Ju H.W., Lee J.M., Moon B.M., Park H.J., Lee O.J., Kim J.H., Kim D.K., Park C.H. (2015). 3D electrospun silk fibroin nanofibers for fabrication of artificial skin. Nanomedicine.

[129] Park Y.R., Ju H.W., Lee J.M., Kim D.K., Lee O.J., Moon B.M., Park H.J., Jeong J.Y., Yeon Y.K., Park C.H. (2016). Three-dimensional electrospun silk-fibroin nanofiber for skin tissue engineering. Int. J. Biol. Macromol..

[130] Holzapfel G.A., Lemaitre J. (2001). Section 10.11—Biomechanics of soft tissue. Handbook of Materials Behavior Models.

[131] Wang Z., Wang Y., Farhangfar F., Zimmer M., Zhang Y. (2012). Enhanced keratinocyte proliferation and migration in co-culture with fibroblasts. PLoS ONE.

[132] Alessandrino A., Chiarini A., Biagiotti M., Dal Prà I., Bassani G.A., Vincoli V., Settembrini P., Pierimarchi P., Freddi G., Armato U. (2019). Three-Layered Silk Fibroin Tubular Scaffold for the Repair and Regeneration of Small Caliber Blood Vessels: From Design to in vivo Pilot Tests. Front. Bioeng. Biotechnol..

[133] Werner S., Krieg T., Smola H. (2007). Keratinocyte-fibroblast interactions in wound healing. J. Investig. Dermatol..

[134] Sun T., McMinn P., Holcombe M., Smallwood R., MacNeil S. (2008). Agent based modelling helps in understanding the rules by which fibroblasts support keratinocyte colony formation. PLoS ONE.

[135] Chummun I., Bhaw-Luximon A., Jhurry D. (2018). Modulating matrix-multicellular response using polysucrose-blended with poly-L-lactide or polydioxanone in electrospun scaffolds for skin tissue regeneration. J. Biomed. Mater. Res. A.

[136] Thayanithy V., O’Hare P., Wong P., Zhao X., Steer C.J., Subramanian S., Lou E. (2017). A transwell assay that excludes exosomes for assessment of tunneling nanotube-mediated intercellular communication. Cell Commun. Signal..

[137] Zhang J., Guan J., Niu X., Hu G., Guo S., Li Q., Xie Z., Zhang C., Wang Y. (2015). Exosomes released from human induced pluripotent stem cells-derived MSCs facilitate cutaneous wound healing by promoting collagen synthesis and angiogenesis. J. Transl. Med..

[138] Zhang W., Bai X., Zhao B., Li Y., Zhang Y., Li Z., Wang X., Luo L., Han F., Zhang J. (2018). Cell-free therapy based on adipose tissue stem cell-derived exosomes promotes wound healing via the PI3K/Akt signaling pathway. Exp. Cell Res..

[139] Chen B., Li Q., Zhao B., Wang Y. (2017). Stem Cell-Derived Extracellular Vesicles as a Novel Potential Therapeutic Tool for Tissue Repair. Stem Cells Transl. Med..

[140] Mota C., Puppi D., Dinucci D., Errico C., Bártolo P., Chiellini F. (2011). Dual-Scale Polymeric Constructs as Scaffolds for Tissue Engineering. Materials.

[141] Mo X.M., Xu C.Y., Kotaki M., Ramakrishna S. (2004). Electrospun P(LLA-CL) nanofiber: A biomimetic extracellular matrix for smooth muscle cell and endothelial cell proliferation. Biomaterials.

[142] Park Y.R., Sultan M.T., Park H.J., Lee J.M., Ju H.W., Lee O.J., Lee D.J., Kaplan D.L., Park C.H. (2018). NF-κB signaling is key in the wound healing processes of silk fibroin. Acta Biomater..

[143] Kochhar D., DeBari M.K., Abbott R.D. (2021). The Materiobiology of Silk: Exploring the Biophysical Influence of Silk Biomaterials on Directing Cellular Behaviors. Front. Bioeng. Biotechnol..

[144] Su N., Gao P.L., Wang K., Wang J.Y., Zhong Y., Luo Y. (2017). Fibrous scaffolds potentiate the paracrine function of mesenchymal stem cells: A new dimension in cell-material interaction. Biomaterials.

[145] Liu T.L., Miao J.C., Sheng W.H., Xie Y.F., Huang Q., Shan Y.B., Yang J.C. (2010). Cytocompatibility of regenerated silk fibroin film: A medical biomaterial applicable to wound healing. J. Zhejiang Univ. Sci. B.

[146] Zhang Y., Sheng R., Chen J., Wang H., Zhu Y., Cao Z., Zhao X., Wang Z., Liu C., Chen Z. (2023). Silk Fibroin and Sericin Differentially Potentiate the Paracrine and Regenerative Functions of Stem Cells through Multiomics Analysis. Adv. Mater..

[147] Chou K.-C., Chen C.-T., Cherng J.-H., Li M.-C., Wen C.-C., Hu S.-I., Wang Y.-W. (2021). Cutaneous Regeneration Mechanism of β-Sheet Silk Fibroin in a Rat Burn Wound Healing Model. Polymers.

[148] French K.C., Antonyak M.A., Cerione R.A. (2017). Extracellular vesicle docking at the cellular port: Extracellular vesicle binding and uptake. Semin. Cell. Dev. Biol..

[149] Wei H., Chen Q., Lin L., Sha C., Li T., Liu Y., Yin X., Xu Y., Chen L., Gao W. (2021). Regulation of exosome production and cargo sorting. Int. J. Biol. Sci..

[150] Li X., Liu Y., Zhang J., You R., Qu J., Li M. (2017). Functionalized silk fibroin dressing with topical bioactive insulin release for accelerated chronic wound healing. Mater. Sci. Eng. C Mater. Biol. Appl..

[151] Fadini G.P., Albiero M., Bonora B.M., Avogaro A. (2011). Angiogenic Abnormalities in Diabetes Mellitus: Mechanistic and Clinical Aspects. J. Clin. Endocrinol. Metab..

