# Supplementary material for: Human Keratinocytes and Fibroblasts Co-Cultured on Silk Fibroin Scaffolds Exosomally Overrelease Angiogenic and Growth Factors
Source: Cells. 2023 Jul 11;12(14):1827. doi: 10.3390/cells12141827 (PMC10378127; doi:10.3390/cells12141827)
Supplement: Supplementary file 1 [file cells-12-01827-s001.zip › cells-2474181-supplementary.pdf]

SUPPLEMENTARY MATERIALS

# Human keratinocytes and fibroblasts co-cultured on silk fibroin scaffolds exosomally overrelease angiogenic and growth factors

**Peng Hu[1], Ubaldo Armato[1,§], Giuliano Freddi[2], Anna Chiarini[1,§,*], and Ilaria Dal Prà[1,§]**

Array 1

Each antibody is spotted in duplicate vertically

| | A | B | C | D | E | F | G | H |
|---|---|---|---|---|---|---|---|---|
| 1, 2 | POS | POS | NEG | NEG | Angiogenin | EGF | ENA-78 (CXCL5) | bFGF |
| 3, 4 | GRO a/b/g | IFN-gamma | IGF-1 | IL-6 | IL-8 (CXCL8) | Leptin | MCP-1 (CCL2) | PDGF-BB |
| 5, 6 | PLGF | RANTES (CCL5) | TGF beta 1 | TIMP-1 | TIMP-2 | TPO | VEGF-A | VEGF-D |
| 7, 8 | BLANK | BLANK | BLANK | BLANK | BLANK | BLANK | NEG | POS |

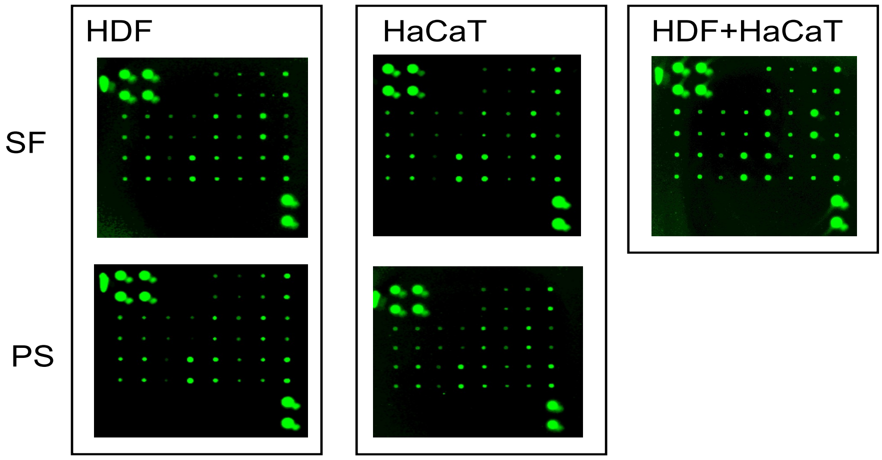


Array 2

Each antibody is spotted in duplicate vertically

| | A | B | C | D | E | F | G | H |
|---|---|---|---|---|---|---|---|---|
| 1, 2 | POS | POS | NEG | NEG | ANGPT1 | ANGPT2 | Angiostatin | Endostatin |
| 3, 4 | GCSF | GM-CSF | I-309 (CCL1) | IL-10 | IL-1 alpha (IL-1 F1) | IL-1 beta (IL-1 F2) | IL-2 | IL-4 |
| 5, 6 | I-TAC (CXCL11) | MCP-3 (CCL7) | MCP-4 (CCL13) | MMP-1 | MMP-9 | PECAM-1 (CD31) | Tie-2 | TNF alpha |
| 7, 8 | uPAR | VEGF R2 | VEGF R3 | BLANK | BLANK | BLANK | BLANK | POS |

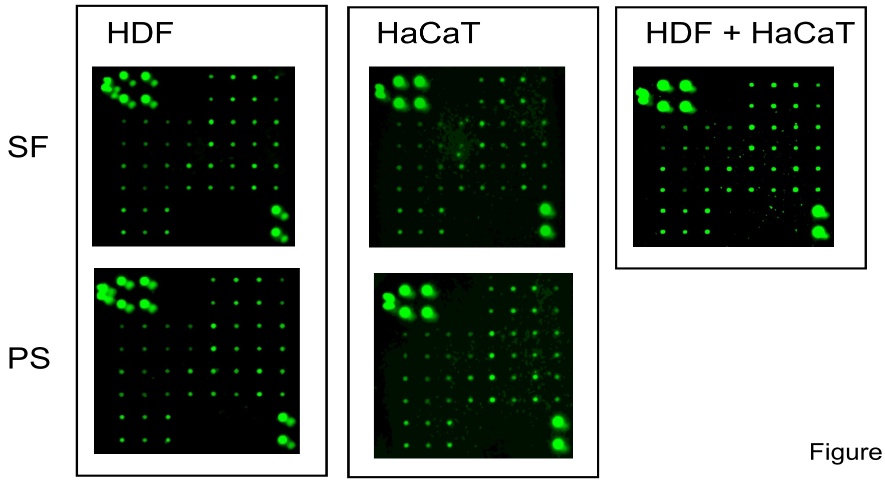


Figure 1S

**Figure S1**. The double-antibody array membranes (each array made of 2 different membranes) showing the AGFs carried by the exosomes released from monocultured or co-cultured HDFs and HaCaTs grown either on C/H-3D-SFnws/ESFN scaffolds or PS. Equal amounts (200 μg) of proteins from the exosomes isolated from conditioned media samples of the various groups were used to conduct the assays.
